# PD-1/PD-L1 inhibitors plus chemotherapy as first-line therapy for advanced or metastatic endometrial cancer: a systematic review and meta-analysis of randomized controlled trials

**DOI:** 10.3389/fimmu.2026.1846834

**Published:** 2026-06-01

**Authors:** Zhilong Huang, Wenwen Li, Minghong Li, Yifu Huang, Yongjiang Lu, Jiahang Hu

**Affiliations:** 1 Guangzhou Women and Children’s Medical Center, Guangzhou Medical University, Guangzhou, China; 2 The First Affiliated Hospital of Guangxi University of Science and Technology, Guangxi University of Science and Technology, Liuzhou, China; 3 Zhongshan Hospital, Fudan University, Shanghai, China; 4 Shanghai Jiao Tong University Medical School Affiliated Ruijin Hospital, Shanghai, China

**Keywords:** chemotherapy, endometrial cancer, meta-analysis, PD-1/PD-L1 inhibitors, trials

## Abstract

**Objective:**

To evaluate the efficacy and safety of programmed death-1/programmed death-ligand 1 (PD-1/PD-L1) inhibitors combined with chemotherapy as first-line treatment for advanced or metastatic endometrial cancer.

**Methods:**

A systematic literature search was conducted in PubMed, Embase, Web of Science, the Cochrane Library, and ClinicalTrials.gov from inception to November 26, 2025. Meta-analyses were performed to pool outcomes including progression-free survival, overall survival, objective response rate, complete response, partial response, stable disease, progressive disease, and treatment-related adverse events.

**Results:**

Ten randomized controlled trials encompassing 4,052 patients were included (combination arm: n=2,109; chemotherapy-alone arm: n=1,943). Compared with chemotherapy alone, the addition of PD-1/PD-L1 inhibitors significantly prolonged median overall survival (43.7 vs. 29.1 months) and median progression-free survival (14.6 vs. 10.2 months). The relative risks (RR) for efficacy endpoints were as follows: complete response, 1.60 (95% CI: 1.25–2.05); partial response, 0.99 (95% CI: 0.88–1.12); stable disease, 0.68 (95% CI: 0.54–0.86); progressive disease, 0.69 (95% CI: 0.45–1.06); and objective response rate, 1.10 (95% CI: 1.02–1.18). The most common treatment-related adverse events were fatigue, alopecia, nausea, peripheral neuropathy, anemia, arthralgia, constipation, and diarrhea.

**Conclusion:**

The combination of PD-1/PD-L1 inhibitors and chemotherapy significantly improves progression-free survival and overall survival in patients with advanced or metastatic endometrial cancer, with the significant OS benefit in dMMR; PFS benefit but no OS benefit in pMMR.

**Systematic Review Registration:**

https://www.crd.york.ac.uk/PROSPERO/view/, identifier CRD420251271038.

## Introduction

Endometrial cancer represents a substantial and growing global health burden among gynecological malignancies, with approximately 417,000 new cases diagnosed annually and a documented annual incidence increase of 1.7%. Factors such as obesity, metabolic syndrome, and increasing life expectancy contribute to its rising prevalence, posing a significant public health challenge (1, 2). In the United States alone, an estimated 69,120 new cases and 13,860 deaths are projected for 2025, accounting for 7% of all new cancer diagnoses in women (3). The current evidence-based standard first-line therapy for primary advanced or recurrent endometrial cancer is combination chemotherapy with carboplatin and paclitaxel (4, 5). Despite this, patient outcomes remain suboptimal, with a median overall survival under three years and a median progression-free survival typically ranging from 10 to 13 months (6, 7).

The advent of immune checkpoint inhibitors has transformed the therapeutic landscape for advanced or recurrent endometrial cancer. This malignancy, particularly the mismatch repair–deficient/microsatellite instability–high (dMMR/MSI-H) subtype, is characterized by a relatively high tumor mutational burden and microsatellite instability, rendering it highly amenable to immunotherapy (8, 9). Mechanistically, the expression of the programmed cell death protein 1 (PD-1) receptor and its ligand (PD-L1) exhibits distinct, subtype-specific patterns, driven by tumor mutational burden-induced T-cell infiltration within the tumor microenvironment (10–12). The consequent upregulation of PD-1/PD-L1 signaling pathways confers particular sensitivity to immune checkpoint blockade in dMMR/MSI-H endometrial cancer (13). Driven by this strong biological rationale and unmet clinical needs, combining PD-1/PD-L1 inhibitors with chemotherapy has emerged as a prominent therapeutic strategy.

This approach has been validated in pivotal randomized controlled trials. For instance, the NRG-GY018 trial demonstrated that in patients with proficient mismatch repair/microsatellite stable (pMMR/MSS) recurrent or metastatic endometrial cancer who derive limited benefit from single-agent immunotherapy, the addition of pembrolizumab to carboplatin-paclitaxel reduced the risk of disease progression or death by 46% (hazard ratio [HR] for progression-free survival = 0.54) (14). The RUBY trial further confirmed a significant survival benefit, reporting a 31% reduction in mortality risk (overall survival HR = 0.69) in the overall intention-to-treat population (14). Additionally, the AtTEnd trial showed superior progression-free survival with atezolizumab plus chemotherapy in both the pMMR/MSS subgroup and the overall cohort, indicating that the benefits of this combination strategy extend across molecular subtypes (15). An increasing number of trials targeting diverse patient populations and survival endpoints continue to evaluate the clinical value of PD-1/PD-L1 inhibitor-based combinations in advanced endometrial cancer (16).

Several meta-analyses published over the past five years have explored the efficacy, particularly the survival benefit, of first-line PD-1/PD-L1 inhibitors combined with chemotherapy in advanced or metastatic endometrial cancer (17–19). While their collective findings suggest a significant improvement in overall survival, these conclusions were limited by the relatively small number of available randomised controlled trials and insufficient pooled data at the time. To address these limitations and incorporate the latest evidence, an updated systematic review and meta-analysis was conducted.

## Methods

### Study design

The present meta-analysis was conducted in accordance with the 2020 Preferred Reporting Items for Systematic Reviews and Meta-Analyses (PRISMA) guidelines (20). The study protocol was registered prospectively in the PROSPERO international registry under the identifier CRD420251271038.

### Search strategy

A comprehensive literature search was undertaken across four electronic databases (PubMed, Embase, Web of Science, and the Cochrane Library) for records published from inception until November 26, 2025. Supplementary searches were conducted on ClinicalTrials.gov to identify ongoing or completed clinical trials. The search strategy was formulated based on the PICOS framework and employed both Medical Subject Headings (MeSH)(“Endometrial”, “Endometrium”) and free-text terms. Core search concepts encompassed “PD-1/PD-L1 inhibitor” and endometrial neoplasms (e.g., “Endometrial”, “Endometrium”). Detailed search records are provided in the **Supplementary Materials**.

Take PubMed as an example:

#1: (((((((((((((((PD-1 inhibitor[Title/Abstract]) OR (Pembrolizumab[Title/Abstract])) OR (Nivolumab[Title/Abstract])) OR (Toripalimab[Title/Abstract])) OR (Tislelizumab[Title/Abstract])) OR (Camrelizumab[Title/Abstract])) OR (GLS-010[Title/Abstract])) OR (Cemiplimab[Title/Abstract])) OR (Sintilimab[Title/Abstract])) OR (Zimberelimab[Title/Abstract])) OR (Prolgolimab[Title/Abstract])) OR (Dostarlimab[Title/Abstract])) OR (PD-L1 inhibitor[Title/Abstract])) OR (Atezolizumab[Title/Abstract])) OR (Durvalumab[Title/Abstract])) OR (Avelumab[Title/Abstract]).

#2: (Endometrial[Title/Abstract]) OR (Endometrium[Title/Abstract]).

#3: #1 AND #2.

### Inclusion and exclusion criteria

Inclusion criteria were as follows: (1) P:Patients diagnosed with advanced or metastatic endometrial cancer; (2) I:Patients in the intervention group received PD-1/L1 inhibitors and chemotherapy as first-line therapy; (3) C:Patients in the controlled group received chemotherapy; (4) O:Outcome focused on safety and efficacy data, including adverse events, overall survival, and progression-free survival; (5)S: Study types: randomized controlled trials.

The exclusion criteria are as follows: (1) Other types of articles, such as case reports, conference, publications, thesis, letters, comments, reviews, meta-analyses, editorials, protocols, etc; (2) Other cancers or diseases; (3) Not relevant; (4) Not first-line treatment; (5) The safety and efficacy data were not reported.

### Selection of articles

The selection process was performed as follows: First, duplicate records were eliminated with EndNote (Version 20; Clarivate Analytics). Subsequently, two reviewers independently screened titles and abstracts against the eligibility criteria. Thereafter, the full texts of potentially relevant studies were assessed. If key safety or efficacy data were not presented in the primary article, associated **Supplementary Materials** were consulted. Outcomes from clinical trials registered on ClinicalTrials.gov were retrieved using their respective NCT identifiers. Discrepancies between reviewers were resolved by consensus or, if needed, by consultation with a third author.

### Data extraction

The data were extracted independently by two reviewers using standardized forms. The extracted information included: (1) Basic study characteristics, including region, trial name, and study phase; (2) Baseline demographics and clinical characteristics of participants, including sample size, age, treatment regimens, and follow-up duration; (3) Safety and efficacy outcomes. Any discrepancies were resolved through consultation with a third investigator.

### Quality assessment

The quality assessment of included trials was performed by two independent reviewers. The risk of bias in randomized controlled trials was appraised using the Cochrane Risk of Bias Tool (21). Any discrepancies were resolved through consensus.

### Statistical analysis

Statistical analysis was performed with Cochrane Review Manager 5.3, Stata 12.0, R software (version 4.5.2, ‘meta’ package) in RStudio (2025.09), and Gradeprofiler. Data extraction from study figures (including Kaplan-Meier curves) was conducted using GetData Graph Digitizer. Individual patient data (IPD) were subsequently reconstructed using the IPDformKM utility, implementing the method described by Guyot et al. (22).

Dichotomous variables were analyzed using risk ratios (RR) and continuous variables using weighted mean differences (WMD), each presented with a 95% confidence interval (CI). Where reported as medians and interquartile ranges, continuous data were transformed to means and standard deviations. Study heterogeneity was assessed with Cochran’s Q test and the I^2^ statistic. A DerSimonian-Laird random-effects model was adopted in cases of considerable heterogeneity (Q-test P value <0.05 or I^2^ >50%); otherwise, a fixed-effects model was used. Statistical significance was set at P value of <0.05.

## Results

### Search results

**Figure 1:**
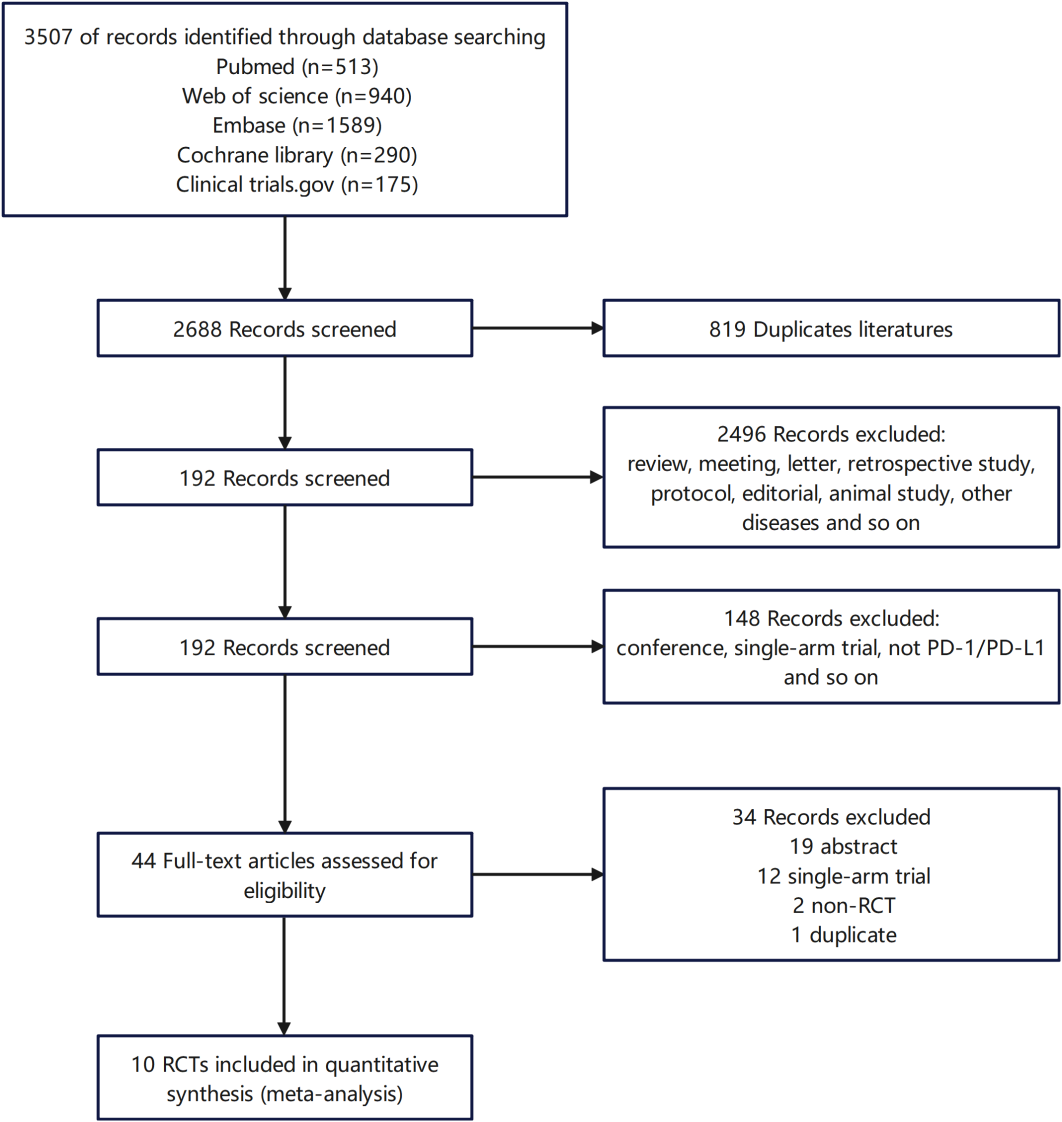
PRISMA flow diagram.

A total of 3,507 studies were initially identified. After removing 819 duplicates primarily via the automatic deduplication function of EndNote software, 2,688 studies remained. Ineligible study types (e.g., review articles, protocols, case reports, animal studies, meta-analyses, and retrospective studies) were excluded, leading to the removal of 2,496 irrelevant publications. Following manual title and abstract screening, an additional 148 articles were excluded. Finally, after full-text review, **Supplementary Material** retrieval, and verification of NCT trial registration numbers, 10 randomized controlled trials met the eligibility criteria (**Figure 1**).

### Patient characteristics

This study included a total of 4,052 eligible patients with advanced or metastatic endometrial cancer, with a median age exceeding 60 years across all studies. Patients were randomized to receive either a combination of PD-1/PD-L1 inhibitors plus chemotherapy (n = 2,109) or chemotherapy alone (n = 1,943). The PD-1/PD-L1 inhibitors used were dostarlimab, durvalumab, atezolizumab, avelumab, and pembrolizumab. Comprehensive baseline characteristics are presented in **Table 1**.

**Table 1:** Baseline characteristics of studies and participants included.

Author, year	Trial name	Trial phase	Region	Sample size (I/C)	Age (I/C, year) Median (range)	Intervention group	Control group	Follow- up time (month, median)
Matthew A.Powell 2025	RUBY	Phase 3	Multinational (19 countries)	Total: 118 I:53 C:65	I: 61 (45–81) C:66 (39–85)	Dostarlimab 500mg + Carboplatin-Paclitaxel	Placebo + Carboplatin-Paclitaxel	36.6
M.R.Mirza 2023	RUBY	Phase 3	Multinational (19 countries)	Total: 494 I:245 C:249	I: 64 (41-81) C:65 (28-85)	Dostarlimab 500 mg IV + Carboplatin AUC 5 + Paclitaxel 175 mg/m² every 3 weeks for 6 cycles, then Dostarlimab 1000 mg IV every 6 weeks for up to 2 years	Placebo IV + Carboplatin AUC 5 + Paclitaxel 175 mg/m² every 3 weeks for 6 cycles, then Placebo IV every 6 weeks for up to 2 years	25.4
M.A.Powell 2024	RUBY	Phase 3	Multinational (19 countries)	Total: 494 I:245 C:249	I: 64 (41-81) C:65 (28–85)	Dostarlimab 500 mg IV + Carboplatin AUC 5 + Paclitaxel 175 mg/m² every 3 weeks for 6 cycles, then Dostarlimab 1000 mg IV every 6 weeks for up to 3 years	Placebo IV + Carboplatin AUC 5 + Paclitaxel 175 mg/m² every 3 weeks for 6 cycles, then Placebo IV every 6 weeks for up to 3 years	37.2
Shin Nishio 2025	DUO-E	Phase 3	Japan	Total: 88 I:26 C:32	I:60 (34–80) C:61 (36–75)	Durvalumab + Carboplatin-Paclitaxel followed by Durvalumab maintenance; Durvalumab+Olaparib maintenance	Carboplatin-Paclitaxel chemotherapy alone	I: 19.2 C: 12.5
Shannon N.Westin 2023	DUO-E	Phase 3	Multinational (22 countries)	Total: 718 I:239 C: 241	I:63 (27-86) C:64 (31-85)	Durvalumab 1500 mg IV + Carboplatin AUC 5 + Paclitaxel 175 mg/m² every 3 weeks for 6 cycles, then Durvalumab 1500 mg IV every 4 weeks ± Olaparib 300 mg PO twice daily for up to 2 years	Placebo IV + Carboplatin AUC 5 + Paclitaxel 175 mg/m² every 3 weeks for 6 cycles, then Placebo IV every 4 weeks for up to 2 years	I: 15.4 C: 12.6
Sandro Pignata 2023	MITO END-3	Phase 2	Italy	Total: 125 I:63 C:62	I:66 (61–72) C:65 (56–70)	Avelumab 10 mg/kg + Carboplatin AUC 5 + Paclitaxel 175 mg/m² every 3 weeks for 6–8 cycles, then Avelumab maintenance every 2 weeks	Carboplatin AUC 5 + Paclitaxel 175 mg/m² every 3 weeks for 6–8 cycles	23·3
Ramez N Eskander 2023	NRG-GY018	Phase 3	United States, Canada, Japan, South Korea	Total: 816 I:405 C:408	pMMR: I: 66 (31–93) C: 65 (29–90) dMMR: I: 67 (38–81) C:66(37–85)	Pembrolizumab 200 mg + Carboplatin AUC 5 + Paclitaxel 175 mg/m² every 3 weeks for 6 cycles, followed by Pembrolizumab 400 mg maintenance every 6 weeks (up to 14 cycles; max 20 cycles )	Placebo 200 mg+ Carboplatin AUC 5 + Paclitaxel 175 mg/m² every 3 weeks for 6 cycles, followed by Placebo 400 mg maintenance every 6 weeks (up to 14 cycles; max 20 cycles )	pMMR: 7.9 dMMR: 12
Ramez N Eskander 2025	NRG GY018	Phase 3	United States, Canada, Japan and Republic of Korea	Total: 810 I: 404 C: 406	pMMR: I: 66 (31–94) C: 66.1(29–91) dMMR: I: 67.2 (39–82) C:66 (37–86)	Pembrolizumab 200 mg + Carboplatin AUC 5 + Paclitaxel 175 mg/m² every 3 weeks for 6 cycles, followed by Pembrolizumab 400 mg maintenance every 6 weeks (up to 14 cycles)	Placebo 200 mg + Carboplatin AUC 5 + Paclitaxel 175 mg/m² every 3 weeks for 6 cycles, followed by Placebo 400 mg maintenance every 6 weeks (up to 14 cycles)	pMMR: 14.4 dMMR: 10
Kenichi Harano 2025	AtTEnd	Phase 3	Asia	Total: 112 I: 67 C: 42	63 (56-68)	Atezolizumab 1200mg + Carboplatin-Paclitaxel followed by Atezolizumab maintenance	Placebo + Carboplatin-Paclitaxel followed by Placebo maintenance	28.3
Nicoletta Colombo 2024	AtTEnd	Phase 3	Multinational (11 countries)	Total: 551 I: 362 C: 189	I: 67 (61-73) C:65 (60-73)	Atezolizumab 1200 mg IV + Carboplatin AUC 5-6 + Paclitaxel 175 mg/m² every 3 weeks	Placebo IV + Carboplatin AUC 5-6 + Paclitaxel 175 mg/m² every 3 weeks	28.3

I, Intervention group, PD-1/PD-L1+chemotherapy.

C, Control group, chemotherapy.

dMMR, deficient mismatch repair.

pMMR, proficient mismatch repair.

### Risk of bias and evidence certainty assessment

**Figure 2:**
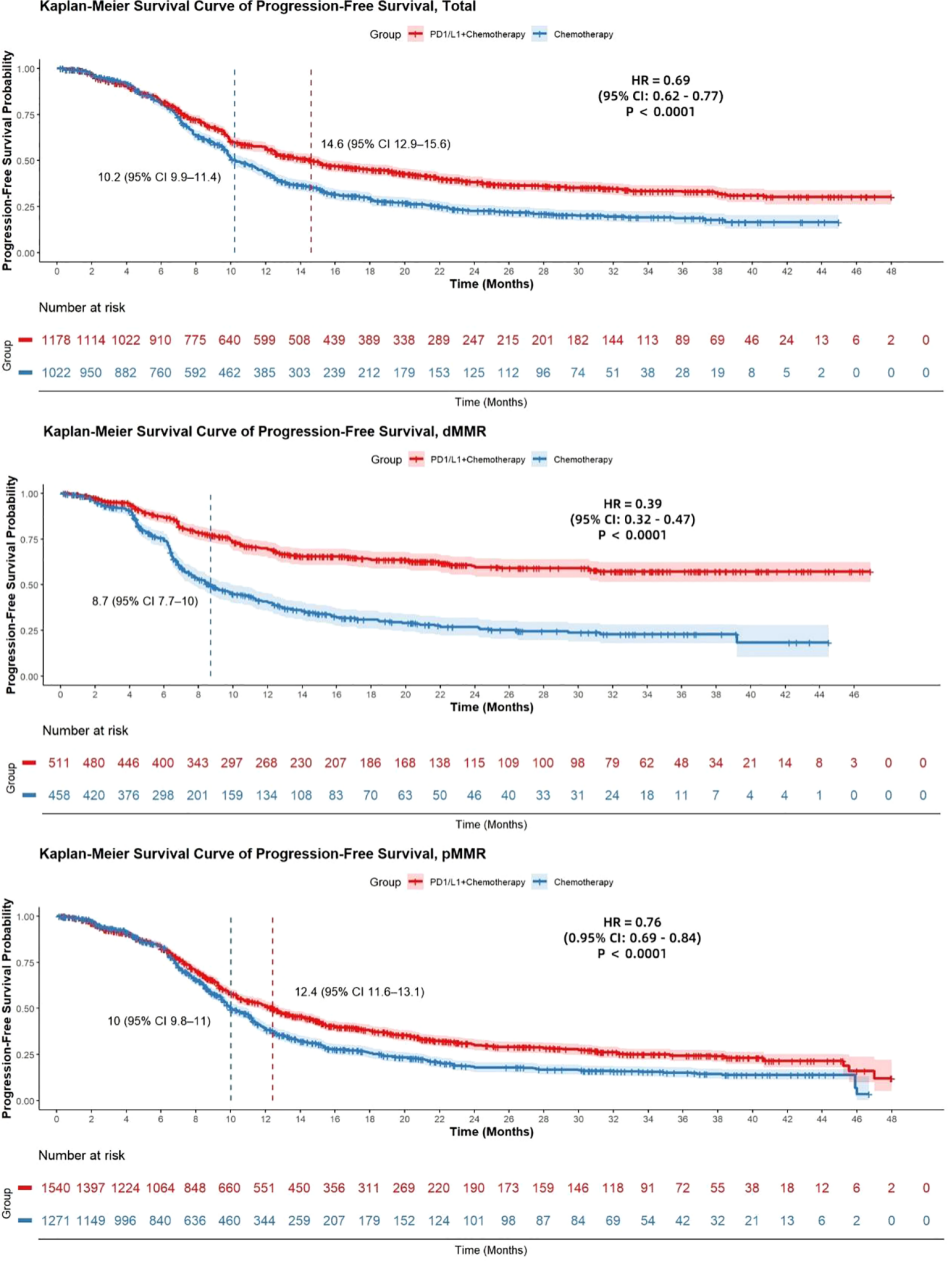
Kaplan-Meier curves for progression-free survival.

Methodological quality of the included trials was assessed using the Cochrane Risk-of-Bias tool, and a summary is shown in **Figure 1**. All trials were judged to have a low risk of bias. The GRADEpro GDT framework was applied to assess the certainty of evidence for selected outcomes. For the comparison between PD-1/PD-L1 inhibitor−based combination therapy and chemotherapy alone, the overall certainty of evidence for any−grade treatment−related adverse events was rated as high (**Figure 2**). Complete GRADE evidence profiles are provided in the **Supplementary Materials**.

### Meta-analysis

Efficacy outcomes are summarized in **Table 2**. The relevant outcomes for advanced and metastatic endometrial cancer patients treated with PD-1/L1 inhibitors combined with chemotherapy are as follows: complete response (1.60, 95%CI, 1.25 to 2.05), partial response (0.99, 95% CI, 0.88 to 1.12), progressive disease (0.69, 95% CI, 0.45 to 1.06), stable disease (0.68, 95% CI, 0.54 to 0.86), and objective response rate (1.10, 95% CI, 1.02 to 1.18). The relevant forest plot can be found in the **Supplementary Materials**.

**Table 2:** Summary of the forest plot results.

Outcomes	No. studies	No. patients	Events	RR (95% CI)	Overall effect size (Z)	Heterogeneity	p-value
						I^2 ^(%)	
Objective response rate	6	2017	1186	1.10(1.02-1.18)P=0.01	2.44	0	0.71
Complete response	5	1594	223	1.60(1.25-2.05)P=0.0002	3.72	3	0.39
Partial response	5	1594	641	0.99(0.88-1.12)P=0.88	0.15	0	0.91
Stable disease	4	1547	247	0.68(0.54-0.86)P=0.001	3.22	0	0.42
Progressive disease	4	1547	82	0.69(0.45-1.06)P=0.09	1.71	0	0.95
Any grade trAEs	9	3817	3760	1.00(0.99-1.00)P=0.13	1.50	0	0.95
Grade ≥ 3 trAEs	8	3038	1795	1.18(1.11-1.25)P<0.00001	5.28	0	0.67
Fatigue	9	3881	2202	1.06(1.00-1.12)P=0.04	2.09	26	0.21
Alopecia	9	3174	1622	1.00(0.93-1.06)P=0.89	0.13	33	0.15
Nausea	10	3939	1868	1.10(0.99-1.23)P=0.09	1.69	75	<0.0001
Neuropathy peripheral	10	3939	1723	0.99(0.92-1.06)P=0.74	0.33	0	0.77
Anemia	10	3939	2008	1.03(0.97-1.09)P=0.39	0.86	32	0.15
Arthralgia	10	3939	1267	0.97(0.89-1.06)P=0.56	0.58	0	0.79
Constipation	10	3939	1512	1.04(0.96-1.13)P=0.30	1.04	30	0.17
Diarrhea	10	3939	1304	1.10(0.95-1.27)P=0.20	1.27	74	<0.0001

Any grade treatment-related adverse events, the result was 1.00 (95% CI, 0.99 to 1.00). For grade≥ 3 treatment-related adverse events rate was found to be 1.18 (95% CI, 1.11 to 1.25) among patients with advanced and metastatic endometrial cancer who received PD-1/L1 inhibitors combined with chemotherapy as first-line treatment. The most common adverse reactions include fatigue 1.06 (95% CI, 1.00 to 1.12), alopecia 1.00 (95% CI, 0.93 to 1.06), nausea 1.10 (95% CI, 0.99 to 1.23), neuropathy peripheral 0.99 (95% CI, 0.92 to 1.06), anemia 1.03 (95% CI, 0.97 to 1.09), arthralgia 0.97 (95% CI, 0.89 to 1.06), constipation 1.04 (95% CI, 0.96 to 1.13), and diarrhea 1.10 (95% CI, 0.95 to 1.27). The detailed results are in Supplement Materials.

Progression−free survival outcomes are shown in **Figure 2**. Compared with chemotherapy alone, first−line PD−1/PD−L1 inhibitor combination therapy significantly reduced the risk of disease progression or death (HR = 0.69, 95% CI: 0.62–0.77; P < 0.001). Subgroup analyses based on mismatch repair status revealed consistent benefits. The magnitude of benefit was greatest in patients with dMMR tumors (HR = 0.39, 95% CI: 0.32–0.47; P < 0.001), while a significant, though more modest, improvement was also observed in the pMMR subgroup (HR = 0.76, 95% CI: 0.69–0.84; P < 0.001). In the intention-to-treat population, median progression-free survival was 14.6 months with combination therapy versus 10.2 months with chemotherapy alone. Among dMMR patients, median progression-free survival was not reached in the combination arm compared with 8.7 months in the control arm; for pMMR patients, median progression-free survival was 12.4 months and 10.0 months, respectively.

**Figure 3:**
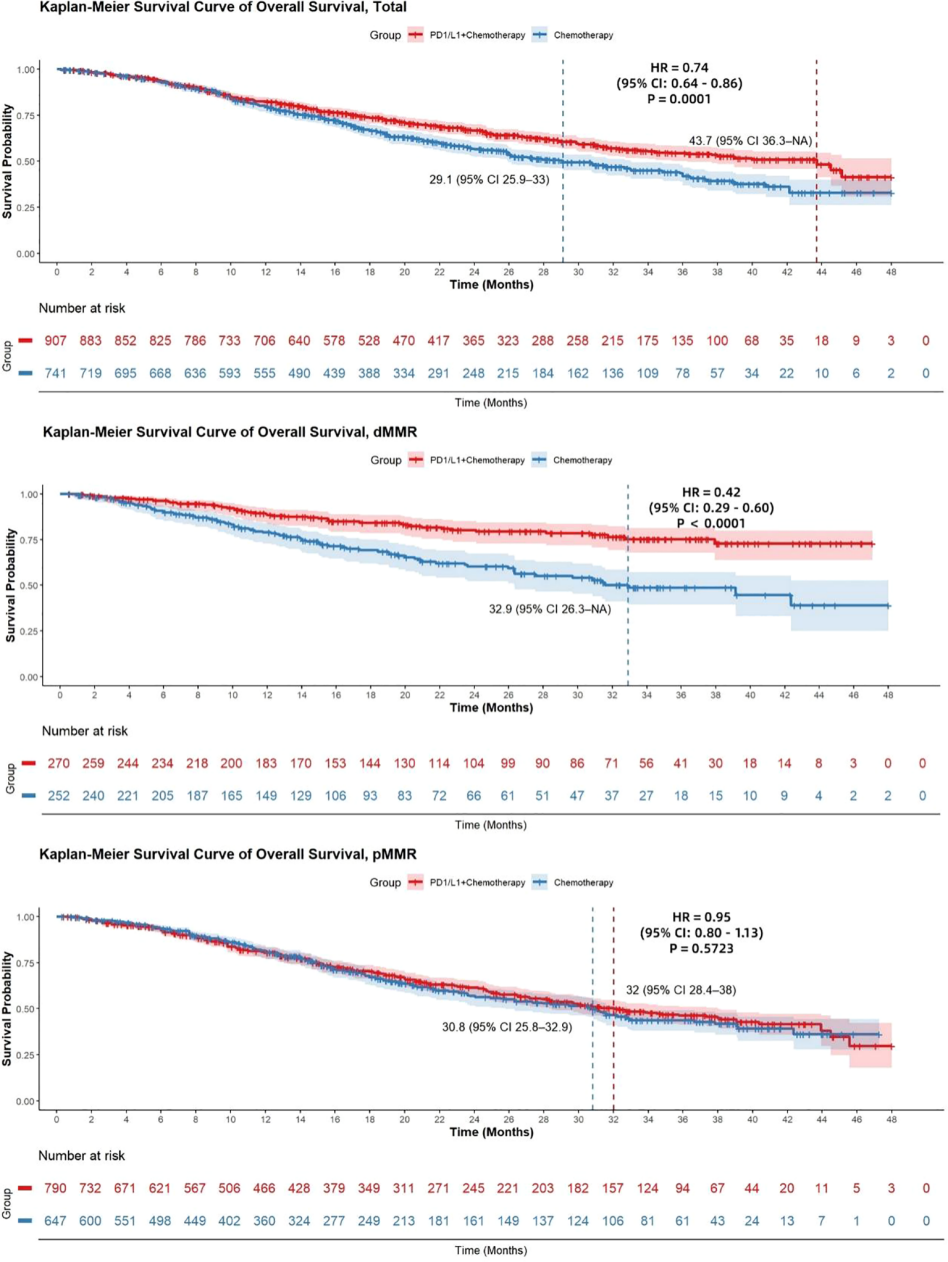
Kaplan-Meier curves for overall survival.

Overall survival results are presented in **Figure 3**. Combination therapy was associated with a significant improvement in overall survival (HR = 0.74, 95% CI: 0.64–0.86; P < 0.001). Median overall survival was 43.7 months in the combination group compared with 29.1 months in the chemotherapy-alone group. When stratified by MMR status, a substantial overall survival benefit was observed in patients with dMMR patients (HR = 0.42, 95% CI: 0.29–0.60; P < 0.001), with median overall survival not reached in the combination arm versus 32.9 months in the control arm. In contrast, for pMMR patients, no statistically significant difference in overall survival was detected between the two treatment groups (HR = 0.95, 95% CI: 0.80–1.13; P = 0.572). Nevertheless, the confidence interval (0.80-1.13) does not exclude a modest potential benefit, and longer follow−up may be warranted.

### Sensitivity analyses and publication bias

Sensitivity analyses were performed for the most outcomes, and the pooled results remained stable after sequential exclusion of individual studies. Publication bias was assessed using funnel plots and Egger’s regression test(P = 0.626), with no significant evidence of bias detected. Further details are available in the **Supplementary Materials**.

## Discussion

This systematic review and meta-analysis of 10 randomized controlled trials demonstrates that first-line PD-1/PD-L1 inhibitor combination therapy represents a superior therapeutic strategy for advanced or metastatic endometrial cancer, conferring significant survival benefits over chemotherapy alone. The combination regimen was associated with a clinically meaningful improvement in overall survival, reducing the risk of death by 26% (HR = 0.74). This translated to a median overall survival of 43.7 months, a 14.6-month extension compared to the 29.1 months observed with chemotherapy alone. The benefit was profoundly greater in the dMMR subgroup, where the risk of death was reduced by 58% (HR = 0.42) and median overall survival was not yet reached. In contrast, no significant overall survival benefit was observed in the pMMR population (HR = 0.95). Similarly, a significant improvement in progression-free survival was observed, with a 31% reduction in the risk of progression or death (HR = 0.69). The median progression-free survival was extended by 4.4 months (14.6 vs. 10.2 months). Again, the magnitude of benefit was biomarker-dependent, with striking risk reductions of 61% in the dMMR subgroup (HR = 0.39) and 24% in the pMMR subgroup (HR = 0.76).

The combination of PD-1/PD-L1 inhibitors with chemotherapy as a first-line treatment for endometrial cancer demonstrates significantly superior overall efficacy compared to chemotherapy alone, with a generally manageable safety profile. In terms of efficacy, the combination therapy significantly improves the complete response rate (RR = 1.60, P < 0.0001), substantially increasing the probability of achieving deep pathological response—a key predictor of improved long-term survival. The objective response rate was also significantly enhanced (RR = 1.10, P = 0.01), indicating that more patients achieved tumor shrinkage. Regarding the dynamics of response, the combination group showed a significantly lower rate of stable disease (RR = 0.68, P < 0.001), suggesting that more patients transitioned from stable disease to objective response, reflecting a robust activation of the antitumor immune response. In terms of safety, the combination therapy was generally well-tolerated. The incidence of any-grade treatment-related adverse events was comparable between the combination and chemotherapy-alone groups (RR = 1.00, P = 0.24), indicating that the addition of immunotherapy did not increase the overall toxicity burden. Common chemotherapy-related toxicities, such as alopecia, anemia, peripheral neuropathy, gastrointestinal events (constipation, diarrhea, nausea), and arthralgia, showed no significant differences between the two groups, suggesting that the addition of immunotherapy did not exacerbate conventional chemotherapy-related toxicities. A modest increase was observed only in the incidence of severe (grade ≥3) treatment-related adverse events (RR = 1.18, P < 0.0001) and a slight but significant increase in fatigue (RR = 1.06, P = 0.04). However, these increases were limited and the events were generally manageable.

Our findings are largely consistent with previous meta-analyses (17–19, 23, 33, 34), collectively affirming the role of PD-1/PD-L1 inhibitors in combination with chemotherapy for first-line treatment. The differential efficacy based on MMR status underscores the critical importance of biomarker stratification, revealing that the dMMR population derives exceptional benefit, while the absolute gain in the pMMR group, though statistically significant for progression-free survival, is more modest. Consistent with previous studies (24), the objective response rate was also significantly enhanced. Similar findings have been documented in prior research (25, 26).

Before the advent of immune checkpoint inhibitors, first-line standard treatment for advanced or recurrent endometrial cancer relied primarily on platinum-based chemotherapy (e.g., carboplatin), usually combined with paclitaxel. Although this regimen achieves objective responses for a certain period, its efficacy has significant limitations: patients generally have a short median progression-free survival, limited long-term survival benefits, and most eventually experience disease progression (27). For patients with pMMR, the tumor microenvironment often exhibits an “immunosuppressive” or “immune-cold” state. Conventional chemotherapy struggles to induce sustained, deep immune responses, resulting in limited treatment options and relatively poorer prognosis (28). In recent years, the application of PD-1/L1 inhibitors combined with chemotherapy has fundamentally transformed this treatment paradigm. Results of the meta-analysis in this study demonstrate that, as first-line treatment, PD-1/L1 inhibitor plus chemotherapy significantly improves overall survival and progression-free survival in patients with advanced/metastatic endometrial cancer compared with chemotherapy alone, with this benefit observed in dMMR subgroup. And for pMMR subgroup, a limited benefit of OS was observed. Underpinning this breakthrough efficacy is the biological basis of the synergistic interaction between immunotherapy and chemotherapy. Chemotherapeutic agents not only exert direct cytotoxic effects but also remodel the tumor immune microenvironment from “immune-cold” to “immune-hot” through multiple mechanisms, including inducing immunogenic cell death of tumor cells, exposing tumor neoantigens, reducing immunosuppressive cells (e.g., regulatory T cells), and upregulating PD-L1 expression on tumor cells (29, 30). Building on this, PD-1/L1 inhibitors relieve the inhibition of T cell activity by tumor cells, thereby allowing for sustained and effective tumor recognition and destruction by the immune system following chemotherapy-induced immune activation, achieving a “1 + 1>2” synergistic effect. Key phase III randomized controlled trials such as KEYNOTE-775, NRG-GY018, and RUBY have validated the universal advantages of this combination strategy across populations with different MMR statuses (14, 31). Notably, in pMMR patients, who typically have a low tumor mutational burden, the combination therapy still yields clinically meaningful improvements in progression-free survival and overall survival. This suggests that the immunomodulatory effects of chemotherapy may create prerequisites for this subset of patients to benefit from immunotherapy (14, 32).

### Advantages of meta-analysis

This study has several notable strengths. First, the search strategy was systematically optimized. By consulting the Medical Subject Headings databases of PubMed, Embase, and the Cochrane Library, two core terms—”Endometrial” and “Endometrium”—were precisely identified, ensuring both comprehensive coverage and high efficiency. Second, the included trials are of high quality and substantial quantity. Strict inclusion criteria were applied to select only high-quality randomized controlled trials, and a total of 10 trials were included—representing the largest number among existing meta-analyses in this field, thereby enhancing the reliability and generalizability of the pooled results. Third, the analysis of safety and efficacy outcomes is comprehensive. On the safety side, we assessed adverse events of any grade and grade ≥3, along with eight common specific adverse reactions. On the efficacy side, we systematically analyzed complete response, partial response, stable disease, progressive disease, and objective response rate, providing a holistic view of the anti-tumor effect. Fourth, the presentation of survival outcomes is clear and interpretable. For overall survival and progression-free survival, Kaplan-Meier curves were reconstructed using the IPDfromKM software package, enabling more accurate data extraction and intuitive visualization, thereby improving clinical interpretability.

### Limitations of meta-analysis

Several limitations should be acknowledged. First, inherent limitations exist in the included randomized controlled trials. Heterogeneity across trials stems from multiple sources: (i) unexplored heterogeneity across different PD−1/PD−L1 inhibitors (dostarlimab, pembrolizumab, atezolizumab, avelumab, durvalumab). Although the PD-1/PD-L1 inhibitors included in this study (dostarlimab, pembrolizumab, atezolizumab, avelumab, and durvalumab) all belong to the class of immune checkpoint inhibitors, they exhibit subtle differences in mechanism of action, dosage, and dosing frequency—for instance, dostarlimab is a PD-1 inhibitor whereas atezolizumab is a PD-L1 inhibitor, and their dosing frequencies differ. Because the sample size for each individual agent within the included studies was limited, subgroup analyses by specific drug could not be performed, precluding a thorough exploration of heterogeneity in efficacy and safety across the different agents. (ii) patients from diverse geographic regions and ethnic backgrounds introduce variability in genetic and clinical characteristics; and (iii) sample sizes varied considerably (88–813 cases), potentially allowing larger trials to disproportionately influence pooled estimates. Second, differences were observed across the included trials in both follow-up duration and the versions of the Common Terminology Criteria for Adverse Events (CTCAE) used for grading. Although the majority of trials had a follow-up duration exceeding 20 months, some had shorter follow-up periods, which may not have adequately captured the delayed therapeutic effect of immunotherapy. Furthermore, variations in the completeness of follow-up and in the handling of missing data could introduce bias into the pooled overall survival estimates. In addition, the CTCAE versions adopted across the studies were inconsistent, and the subtle differences in grading definitions for certain adverse events between versions may contribute to heterogeneity in the reported incidence rates of adverse events. Third, the observed PFS improvement in the pMMR/MSS subgroup in our meta-analysis cannot be entirely attributed to PD-1/PD-L1 blockade plus chemotherapy; the additional maintenance PARP inhibitor may partially contribute to the favourable survival outcome. This confounding effect should be taken into account when interpreting the magnitude of treatment benefit, especially for clinical decision-making in pMMR/MSS patients. Fourth, in patients with pMMR tumors, no significant overall survival benefit was observed (HR = 0.95, P = 0.572). This negative finding carries important clinical implications, and the underlying mechanisms warrant further in-depth exploration from the following perspectives: (i) Molecular mechanisms: pMMR tumor cells retain intact DNA repair function, resulting in a low tumor mutational burden (TMB) and consequently a paucity of tumor-associated antigens. This limits the effective activation of the host antitumor immune response, thereby attenuating the synergistic effect of PD-1/PD-L1 inhibitors combined with chemotherapy. (ii) Clinical characteristics: Patients with pMMR tumors may harbor a more complex tumor microenvironment—characterized by, for example, increased infiltration of immunosuppressive cells and aberrant cytokine secretion—which compromises the efficacy of immunotherapy. (iii) Treatment-related factors: In the included studies, heterogeneity in chemotherapy regimens, immunotherapy dosing, and treatment duration among pMMR patients may have potentially influenced survival outcomes. Fifth, due to insufficient raw data, subgroup analyses stratified by PD-L1 expression or histological subtype could not be performed. PD-L1 expression status (e.g., positive vs. negative, expression cut-off thresholds) and histological subtype (e.g., endometrioid carcinoma, serous carcinoma) are key factors influencing the efficacy of PD-1/PD-L1 inhibitors. However, among the 10 RCTs included in this analysis, some studies did not provide detailed data on PD-L1 expression or the specific distribution of histological subtypes, and the PD-L1 detection methods and positivity criteria were inconsistent across studies. These limitations precluded the extraction of valid data for the relevant subgroup analyses. Sixth, the statistical power of the test for publication bias was limited. Only 10 RCTs were included in this analysis, whereas Egger’s test generally requires a minimum of 10–15 studies to ensure adequate statistical power. Therefore, the results of the publication bias assessment in this study may carry certain limitations, and the possibility of publication bias cannot be entirely excluded. To mitigate this limitation as much as possible, funnel plots have been provided in the **Supplementary Materials** to visually illustrate the publication bias. Furthermore, given the limited number of RCTs investigating first-line PD-1/PD-L1 inhibitor plus chemotherapy for advanced or metastatic endometrial cancer, the sample size of the currently included studies does not meet the optimal power requirement for Egger’s test. As more relevant studies become available in the future, the sample size can be expanded, thereby enhancing the reliability of the publication bias assessment.

### Limitations of published data meta-analysis

Beyond the issues of heterogeneity and limited study numbers already discussed, our work carries inherent limitations that stem from its reliance on published aggregate data. First, several of the subgroup analyses included in this meta-analysis—most notably the stratification by MMR status—were reported as secondary or exploratory endpoints in the original trials. Such subgroup analyses are frequently *post hoc* and may be substantially underpowered to detect true treatment–subgroup interactions. Consequently, the apparent absence of an effect in certain strata could reflect insufficient statistical power rather than genuine biological non-responsiveness.

Second, aggregated data do not allow adjustment for patient-level confounders. Factors known to influence immunotherapy outcomes—such as PD-L1 expression levels, number and type of prior therapies, histological subtype, and performance status—cannot be accounted for when only group-level summaries are available. The resulting ecological bias may distort the estimated subgroup effects and precludes a robust assessment of effect modification.

Third, it is important to recognize that a meta-analysis of published results cannot replace an individual participant data (IPD) meta-analysis. IPD meta-analysis remains the gold standard for investigating subgroup effects because it enables time-to-event analyses with proper adjustment for baseline covariates and direct testing of treatment-by-covariate interactions. Without access to individual-level data, the subgroup analyses presented here must be interpreted with caution.

Finally, our observation that overall survival benefit was not statistically significant in the pMMR subgroup deserves particular restraint. This finding may be driven by underpowered subgroup strata, by a diluting effect of crossover in the original trials, or by a true lack of efficacy. The published aggregate data do not permit us to disentangle these possibilities. Therefore, any recommendation to regard a particular treatment strategy as “standard” specifically for pMMR tumors would be premature based on the current evidence. Instead, our data highlight the critical need for a prospective IPD meta-analysis—or large, biomarker-stratified randomized trials—to definitively address this question.

### Cost-effectiveness and clinical value by molecular subgroup

Our risk-benefit analysis highlights important differences in the clinical value of combination therapy across molecular subgroups, which have critical implications for cost-effectiveness and clinical practice.

In dMMR/MSI-H patients, the combination regimen yielded a robust PFS benefit with a NNT of 4 patients, meaning only 4 patients need to be treated to achieve one additional 12-month progression-free survivor. This highly favourable efficacy signal far outweighs the increased toxicity burden (NNH = 10), supporting combination therapy as a high-value, cost-effective standard of care in this population.

In pMMR/MSS patients, the PFS benefit was modest (NNT = 8), and no significant overall survival benefit was observed. The risk-benefit ratio is narrow, with a NNT of 8 versus a NNH of 10, indicating that the modest PFS gain is offset by increased toxicity. Combined with the high acquisition costs of PD-1/PD-L1 inhibitors, universal use of combination therapy is unlikely to be cost-effective in the pMMR/MSS population. Therefore, combination therapy cannot be recommended as a standard of care for all pMMR/MSS patients. Instead, it should be reserved for carefully selected individuals, such as those with very high risk of early progression or poor prognostic features, and only after thorough shared decision-making that explicitly balances the modest PFS benefit, increased toxicity, financial burden, and absence of proven long-term survival benefit.

The combination of PD−1/PD−L1 inhibitors with chemotherapy should be considered a standard first−line option for advanced or metastatic endometrial cancer. MMR status is essential for treatment decision−making: dMMR patients derive profound survival benefits, while pMMR patients still achieve clinically meaningful improvements, particularly in progression-free-survival. For pMMR, PFS was improved, but OS was not; therefore, the combination cannot be routinely recommended as a standard in pMMR. And treatment decisions should be individualised. The safety profile is manageable, with no significant increase in most chemotherapy−related toxicities. Clinicians should monitor for grade ≥3 adverse events and fatigue, though these are generally controlled.

Future studies should prioritize: (i) large−scale, multicenter randomized controlled trials with extended follow−up to confirm long−term efficacy and monitor late−onset adverse events; (ii) head−to−head comparisons of different PD−1/PD−L1 inhibitors to identify optimal agents and regimens; (iii) better standardization of outcome definitions across trials; (iv) more detailed subgroup reporting (e.g., by PD−L1 expression levels, histologic subtypes, prior treatment lines) to enable precise stratification; and (v) investigation of combination strategies in underrepresented populations (e.g., non−Asian, elderly, or comorbid patients) to enhance generalizability.

## Conclusion

In summary, this meta-analysis confirms that first-line therapy with PD-1/PD-L1 inhibitors combined with chemotherapy significantly improves both progression-free and overall survival in patients with advanced or recurrent endometrial cancer compared to chemotherapy alone. The benefit is most pronounced in the dMMR subgroup, which derives exceptional survival advantage, while a significant but more modest progression-free survival benefit is also observed in the pMMR population. However, this enhanced efficacy is accompanied by a quantifiable increase in the incidence of grade ≥3 treatment-related adverse events. These findings underscore the dual importance of biomarker-driven patient selection to maximize therapeutic benefit and vigilant management of toxicity in clinical practice. To further refine patient stratification and solidify the evidence base, future large-scale, multicenter randomized controlled trials with extended follow-up are warranted.

## Data Availability

The original contributions presented in the study are included in the article/**Supplementary Material**. Further inquiries can be directed to the corresponding authors.

## References

[1] Sung H Ferlay J Siegel RL Laversanne M Soerjomataram I Jemal A . Global cancer statistics 2020: Globocan estimates of incidence and mortality worldwide for 36 cancers in 185 countries. CA: A Cancer J For Clin. (2021) 71:209–49. doi: 10.3322/caac.21660. PMID: 33538338

[2] Aune D Navarro Rosenblatt DA Chan DS Vingeliene S Abar L Vieira AR . Anthropometric factors and endometrial cancer risk: A systematic review and dose-response meta-analysis of prospective studies. Ann Oncol. (2015) 26:1635–48. doi: 10.1093/annonc/mdv142. PMID: 25791635

[3] Siegel RL Kratzer TB Giaquinto AN Sung H Jemal A . Cancer statistics, 2025. CA: A Cancer J For Clin. (2025) 75:10–45. doi: 10.3322/caac.21871. PMID: 39817679 PMC11745215

[4] Marchetti M Ferrari J Vezzaro T Masatti L Tasca G Maggino T . The role of immunotherapy in mmr-deficient endometrial carcinoma: State of the art and future perspectives. J Clin Med. (2024) 13(23):7041. doi: 10.3390/jcm13237041. PMID: 39685500 PMC11642574

[5] Włodarczyk-Ciekańska K Kwiatkowska-Makuch A Pawłowska-Łachut A Skiba W Suszczyk D Kotarski J . Assessment of the pd-1/pd-l1/pd-l2 immune checkpoints pathway in endometrial cancer and its clinical significance. Cancers (Basel). (2025) 17(21):3485. doi: 10.3390/cancers17213485. PMID: 41228278 PMC12607983

[6] Miller DS Filiaci VL Mannel RS Cohn DE Matsumoto T Tewari KS . Carboplatin and paclitaxel for advanced endometrial cancer: Final overall survival and adverse event analysis of a phase iii trial (nrg oncology/gog0209). J Clin Oncol Off J Am Soc Clin Oncol. (2020) 38:3841–50. doi: 10.1200/jco.20.01076. PMID: 33078978 PMC7676887

[7] Kalampokas E Giannis G Kalampokas T Papathanasiou AA Mitsopoulou D Tsironi E . Current approaches to the management of patients with endometrial cancer. Cancers (Basel). (2022) 14(18):4500. doi: 10.3390/cancers14184500. PMID: 36139659 PMC9497194

[8] André T Berton D Curigliano G Sabatier R Tinker AV Oaknin A . Antitumor activity and safety of dostarlimab monotherapy in patients with mismatch repair deficient solid tumors: A nonrandomized controlled trial. JAMA Netw Open. (2023) 6:e2341165. doi: 10.1001/jamanetworkopen.2023.41165. PMID: 37917058 PMC10623195

[9] Riedinger CJ Esnakula A Haight PJ Suarez AA Chen W Gillespie J . Characterization of mismatch-repair/microsatellite instability-discordant endometrial cancers. Cancer. (2024) 130:385–99. doi: 10.1002/cncr.35030. PMID: 37751191 PMC10843110

[10] O'Malley DM Bariani GM Cassier PA Marabelle A Hansen AR De Jesus Acosta A . Pembrolizumab in patients with microsatellite instability-high advanced endometrial cancer: Results from the keynote-158 study. J Clin Oncol Off J Am Soc Clin Oncol. (2022) 40:752–61. doi: 10.1200/jco.21.01874. PMID: 34990208 PMC8887941

[11] O'Malley DM Bariani GM Cassier PA Marabelle A Hansen AR De Jesus Acosta A . Health-related quality of life with pembrolizumab monotherapy in patients with previously treated advanced microsatellite instability high/mismatch repair deficient endometrial cancer in the keynote-158 study. Gynecol Oncol. (2022) 166:245–53. doi: 10.1016/j.ygyno.2022.06.005. PMID: 35835611

[12] Oaknin A Gilbert L Tinker AV Brown J Mathews C Press J . Safety and antitumor activity of dostarlimab in patients with advanced or recurrent dna mismatch repair deficient/microsatellite instability-high (dmmr/msi-h) or proficient/stable (mmrp/mss) endometrial cancer: Interim results from garnet-a phase i, single-arm study. J Immunother Cancer. (2022) 10(1):e003777. doi: 10.1136/jitc-2021-003777. PMID: 35064011 PMC8785197

[13] Oaknin A Pothuri B Gilbert L Sabatier R Brown J Ghamande S . Safety, efficacy, and biomarker analyses of dostarlimab in patients with endometrial cancer: Interim results of the phase i garnet study. Clin Cancer Res. (2023) 29:4564–74. doi: 10.1158/1078-0432.ccr-22-3915. PMID: 37363992 PMC10643997

[14] Eskander RN Sill MW Beffa L Moore RG Hope JM Musa FB . Pembrolizumab plus chemotherapy in advanced or recurrent endometrial cancer: Overall survival and exploratory analyses of the nrg gy018 phase 3 randomized trial. Nat Med. (2025) 31:1539–46. doi: 10.1038/s41591-025-03566-1. PMID: 40044930 PMC12851417

[15] Powell MA Bjørge L Willmott L Novák Z Black D Gilbert L . Overall survival in patients with endometrial cancer treated with dostarlimab plus carboplatin-paclitaxel in the randomized engot-en6/gog-3031/ruby trial. Ann Oncol. (2024) 35:728–38. doi: 10.1016/j.annonc.2024.05.546. PMID: 38866180

[16] Colombo N Biagioli E Harano K Galli F Hudson E Antill Y . Atezolizumab and chemotherapy for advanced or recurrent endometrial cancer (attend): A randomised, double-blind, placebo-controlled, phase 3 trial. Lancet Oncol. (2024) 25:1135–46. doi: 10.1016/s1470-2045(24)00334-6. PMID: 39102832

[17] Ren J Wang J Wang Y Yang D Sheng J Zhu S . Efficacy and safety of pd-1/pd-l1 inhibitors in advanced or recurrent endometrial cancer: A meta-analysis with trial sequential analysis of randomized controlled trials. Front Immunol. (2025) 16:1521362. doi: 10.3389/fimmu.2025.1521362. PMID: 39958346 PMC11825832

[18] Villacampa G Eminowicz G Navarro V Carità L García-Illescas D Oaknin A . Immunotherapy and parp inhibitors as first-line treatment in endometrial cancer: A systematic review and network meta-analysis. Eur J Cancer. (2025) 220:115329. doi: 10.1016/j.ejca.2025.115329. PMID: 40031426

[19] Bartoletti M Montico M Lorusso D Mazzeo R Oaknin A Musacchio L . Incorporation of anti-pd1 or anti pd-l1 agents to platinum-based chemotherapy for the primary treatment of advanced or recurrent endometrial cancer. A meta-analysis. Cancer Treat Rev. (2024) 125:102701. doi: 10.1016/j.ctrv.2024.102701. PMID: 38422895

[20] Page MJ McKenzie JE Bossuyt PM Boutron I Hoffmann TC Mulrow CD . The prisma 2020 statement: An updated guideline for reporting systematic reviews. Systematic Rev. (2021) 10:89. doi: 10.1186/s13643-021-01626-4. PMID: 33781348 PMC8008539

[21] Crocker TF Lam N Jordão M Brundle C Prescott M Forster A . Risk-of-bias assessment using cochrane's revised tool for randomized trials (rob 2) was useful but challenging and resource-intensive: Observations from a systematic review. J Clin Epidemiol. (2023) 161:39–45. doi: 10.1016/j.jclinepi.2023.06.015. PMID: 37364620

[22] Liu N Zhou Y Lee JJ . Ipdfromkm: Reconstruct individual patient data from published kaplan-meier survival curves. BMC Med Res Methodol. (2021) 21:111. doi: 10.1186/s12874-021-01308-8. PMID: 34074267 PMC8168323

[23] de Moraes FCA Pasqualotto E Lopes LM Cavalcanti Souza ME de Oliveira Rodrigues ALS de Almeida AM . Pd-1/pd-l1 inhibitors plus carboplatin and paclitaxel compared with carboplatin and paclitaxel in primary advanced or recurrent endometrial cancer: A systematic review and meta-analysis of randomized clinical trials. BMC Cancer. (2023) 23(1):1166. doi: 10.1186/s12885-023-11654-z. PMID: 38031003 PMC10688003

[24] de Liz CD Maia M Alves ACF da Silva IMM Oliveira JP de Oliveira ACF . Chemotherapy plus pd-1/pd-l1 inhibitor versus chemotherapy alone in first-line treatment for recurrent or advanced endometrial cancer: A systematic review and meta-analysis of randomized controlled trials. J Clin Oncol. (2024) 42:e17597–e17597. doi: 10.1200/jco.2024.42.16_suppl.e17597. PMID: 42148471

[25] Auranen A Powell MA Sukhin V Landrum LM Ronzino G Buscema J . Safety of dostarlimab in combination with chemotherapy in patients with primary advanced or recurrent endometrial cancer in a phase iii, randomized, placebo-controlled trial (engot-en6-nsgo/gog-3031/ruby). Ther Adv Med Oncol. (2024) 16:17588359241277656. doi: 10.1177/17588359241277656. PMID: 39346117 PMC11439170

[26] Westin SN Moore K Chon HS Lee JY Thomes Pepin J Sundborg M . Durvalumab plus carboplatin/paclitaxel followed by maintenance durvalumab with or without olaparib as first-line treatment for advanced endometrial cancer: The phase iii duo-e trial. J Clin Oncol Off J Am Soc Clin Oncol. (2024) 42:283–99. doi: 10.1200/jco.23.02132. PMID: 37864337 PMC10824389

[27] Fleming GF Brunetto VL Cella D Look KY Reid GC Munkarah AR . Phase iii trial of doxorubicin plus cisplatin with or without paclitaxel plus filgrastim in advanced endometrial carcinoma: A gynecologic oncology group study. J Clin Oncol Off J Am Soc Clin Oncol. (2004) 22:2159–66. doi: 10.1200/jco.2004.07.184. PMID: 15169803

[28] Oaknin A Bosse TJ Creutzberg CL Giornelli G Harter P Joly F . Endometrial cancer: Esmo clinical practice guideline for diagnosis, treatment and follow-up. Ann Oncol. (2022) 33:860–77. doi: 10.1016/j.annonc.2022.05.009. PMID: 35690222

[29] Galluzzi L Buqué A Kepp O Zitvogel L Kroemer G . Immunogenic cell death in cancer and infectious disease. Nat Rev Immunol. (2017) 17:97–111. doi: 10.1038/nri.2016.107. PMID: 27748397

[30] Pfirschke C Engblom C Rickelt S Cortez-Retamozo V Garris C Pucci F . Immunogenic chemotherapy sensitizes tumors to checkpoint blockade therapy. Immunity. (2016) 44:343–54. doi: 10.1016/j.immuni.2015.11.024. PMID: 26872698 PMC4758865

[31] Eskander RN Sill MW Beffa L Moore RG Hope JM Musa FB . Pembrolizumab plus chemotherapy in advanced endometrial cancer. N Engl J Med. (2023) 388:2159–70. doi: 10.1056/NEJMoa2302312. PMID: 36972022 PMC10351614

[32] Mirza MR Chase DM Slomovitz BM Christensen RD Novak Z Black D . Dostarlimab for primary advanced or recurrent endometrial cancer. N Engl J Med. (2023) 388:2145–58. doi: 10.1056/NEJMoa2216334. PMID: 36972026

[33] Li YT Li SX Liang J . Efficacy and safety of immune checkpoint inhibitors combined with chemotherapy or tyrosine kinase inhibitors in advanced endometrial cancer: A systematic review and meta-analysis. Gynecologic Obstetric Invest. (2025) 90:241–54. doi: 10.1159/000541617. PMID: 39496257

[34] Gouveia MC Bonadio RC Neto FL Trento MMS Cunha MT Scaranti M . Pd-1 or pd-l1 inhibitors in addition to first-line chemotherapy for endometrial cancer: An extracted individual patient data meta-analysis. ecancermedicalscience. (2025) 19:1884. doi: 10.3332/ecancer.2025.1884. PMID: 40496311 PMC12149239

