# Supplementary material for: PD-1/PD-L1 inhibitors plus chemotherapy as first-line therapy for advanced or metastatic endometrial cancer: a systematic review and meta-analysis of randomized controlled trials
Source: Front Immunol. 2026 Jun 1;17:1846834. doi: 10.3389/fimmu.2026.1846834 (PMC13265514; doi:10.3389/fimmu.2026.1846834)
Supplement: Supplementary file 1 [file Table1.docx]

**Methods:** Search strategy

**eFigure 1.** Summary of risks of bias of all included studies

**eFigure 2.** The GRADE Rating for the comparisons of PD-1 /PD-L1 vs control groups in all included studies

**eFigure 3.** Forest plot of Complete Response Rate

**eFigure 4.** Forest plot of Objective Response Rate

**eFigure 5.** Forest plot of Progressive Disease Rate

**eFigure 6.** Forest plot of Partial Response Rate

**eFigure 7.** Forest plot of Stable desease Rate

**eFigure 8.** Forest plot of Alopecia

**eFigure 9.** Forest plot of Anemia

**eFigure 10.** Forest plot of Any grade ≥3 TRAE

**eFigure 11.** Forest plot of Any TRAE

**eFigure 12.** Forest plot of Arthralgia

**eFigure 13.** Forest plot of Constipation

**eFigure 14.** Forest plot of Diarrhea

**eFigure 15.** Forest plot of Fatigue

**eFigure 16.** Forest plot of Nausea

**eFigure 17.** Forest plot of Neuropathy peripheral

**eFigure 18.** Sensitivity analysis of Alopecia

**eFigure 19.** Sensitivity analysis of Anemia

**eFigure 20.** Sensitivity analysis of Any grade ≥3 TRAE

**eFigure 21.** Sensitivity analysis of Any TRAE

**eFigure 22.** Sensitivity analysis of Arthralgia

**eFigure 23.** Sensitivity analysis of Constipation

**eFigure 24.** Sensitivity analysis of Diarrhea

**eFigure 25.** Sensitivity analysis of Fatigue

**eFigure 26.** Sensitivity analysis of Nausea

**eFigure 27.** Sensitivity analysis of Neuropathy peripheral

**eFigure 28.** Sensitivity analysis of ORR

**eFigure 29.** Sensitivity analysis of CR

**eFigure 30.** Sensitivity analysis of PD

**eFigure 31.** Sensitivity analysis of PR

**eFigure 32.** Sensitivity analysis of SD

**eFigure 33.** Funnel plot of Complete Response Rate

**eFigure 34.** Funnel plot of Objective Response Rate

**eFigure 35.** Funnel plot of Progressive Disease Rate

**eFigure 36.** Funnel plot of Partial Response Rate

**eFigure 37.** Funnel plot of Stable desease Rate

**eFigure 38.** Funnel plot of Alopecia

**eFigure 39.** Funnel plot of Anemia

**eFigure 40.** Funnel plot of Any grade ≥3 TRAE

**eFigure 41.** Funnel plot of Any TRAE

**eFigure 42.** Funnel plot of Arthralgia

**eFigure 43.** Funnel plot of Constipation

**eFigure 44.** Funnel plot of Diarrhea

**eFigure 45.** Funnel plot of Fatigue

**eFigure 46.** Funnel plot of Nausea

**eFigure 47.** Funnel plot of Neuropathy peripheral

**eFigure 48.** Forest plot of PFS

**eFigure 49.** Sensitivity analysis of PFS

**eFigure 50.** Funnel plot of PFS

**eFigure 51.** Forest plot of OS

**eFigure 52.** Sensitivity analysis of OS

**eFigure 53.** Funnel plot of OS

**Note:**Complete definitions of outcome

ClinicalTrials.gov

Endometrial OR Endometrium | PD-1 inhibitor OR Pembrolizumab OR Nivolumab OR Toripalimab OR Tislelizumab OR Camrelizumab OR GLS-010 OR Cemiplimab OR Sintilimab OR Zimberelimab OR Prolgolimab OR Dostarlimab OR PD-L1 inhibitor OR Atezolizumab OR Durvalumab OR Avelumab 175

| PubMed | | |
| --- | --- | --- |
| No. | Query | Results |
| #1 | (((((((((((((((PD-1 inhibitor[Title/Abstract]) OR (Pembrolizumab[Title/Abstract])) OR (Nivolumab[Title/Abstract])) OR (Toripalimab[Title/Abstract])) OR (Tislelizumab[Title/Abstract])) OR (Camrelizumab[Title/Abstract])) OR (GLS-010[Title/Abstract])) OR (Cemiplimab[Title/Abstract])) OR (Sintilimab[Title/Abstract])) OR (Zimberelimab[Title/Abstract])) OR (Prolgolimab[Title/Abstract])) OR (Dostarlimab[Title/Abstract])) OR (PD-L1 inhibitor[Title/Abstract])) OR (Atezolizumab[Title/Abstract])) OR (Durvalumab[Title/Abstract])) OR (Avelumab[Title/Abstract]) | 30536 |
| #2 | (Endometrial[Title/Abstract]) OR (Endometrium[Title/Abstract]) | 95661 |
| #3 | #1 AND #2 | 513 |

| cochrane library | | |
| --- | --- | --- |
| No. | Query | Results |
| #1 | (PD-1 inhibitor OR Pembrolizumab OR Nivolumab OR Toripalimab OR Camrelizumab OR GLS-010 OR Cemiplimab OR Sintilimab OR Zimberelimab OR Prolgolimab OR Dostarlimab OR PD-L1 inhibitor OR Atezolizumab OR Durvalumab OR Avelumab):ab,ti,kw | 12545 |
| #2 | (Endometrial OR Endometrium):ab,ti,kw | 10658 |
| #3 | #1 AND #2 | 290 |

| Embase | | |
| --- | --- | --- |
| No. | Query | Results |
| #1 | 'pd-1 inhibitor' OR (('pd 1'/exp OR 'pd 1') AND ('inhibitor'/exp OR inhibitor)) OR pembrolizumab:ti,ab,kw OR nivolumab:ti,ab,kw OR toripalimab:ti,ab,kw OR tislelizumab:ti,ab,kw OR camrelizumab:ti,ab,kw OR 'gls 010':ti,ab,kw OR cemiplimab:ti,ab,kw OR sintilimab:ti,ab,kw OR zimberelimab:ti,ab,kw OR prolgolimab:ti,ab,kw OR dostarlimab:ti,ab,kw OR 'pd-l1 inhibitor':ti,ab,kw OR atezolizumab:ti,ab,kw OR durvalumab:ti,ab,kw OR avelumab:ti,ab,kw | 90196 |
| #2 | Endometrial:ab,ti,kw OR Endometrium:ab,ti,kw | 145202 |
| #3 | #1 AND #2 | 1589 |

| Web of science | | |
| --- | --- | --- |
| No. | Query | Results |
| #1 | TS=(PD-1 inhibitor OR Pembrolizumab OR Nivolumab OR Toripalimab OR Tislelizumab OR Camrelizumab OR GLS-010 OR Cemiplimab OR Sintilimab OR Zimberelimab OR Prolgolimab OR Dostarlimab OR PD-L1 inhibitor OR Atezolizumab OR Durvalumab OR Avelumab) | 71644 |
| #2 | TS=(Endometrial OR Endometrium) | 76187 |
| #3 | #1 AND #2 | 940 |

**eFigure 1.** Summary of risks of bias of all included studies


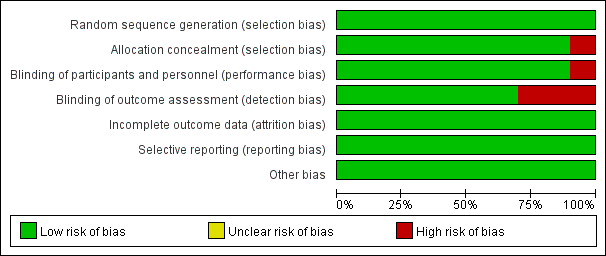


**eFigure 2.** The GRADE Rating for the comparisons of PD-1 /PD-L1 vs control groups in all included studies


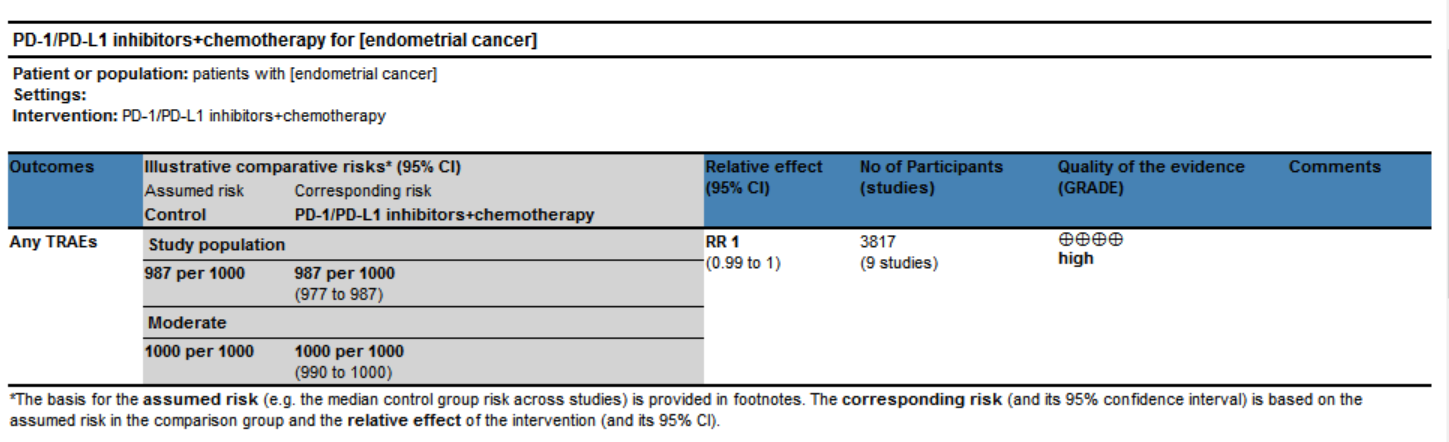


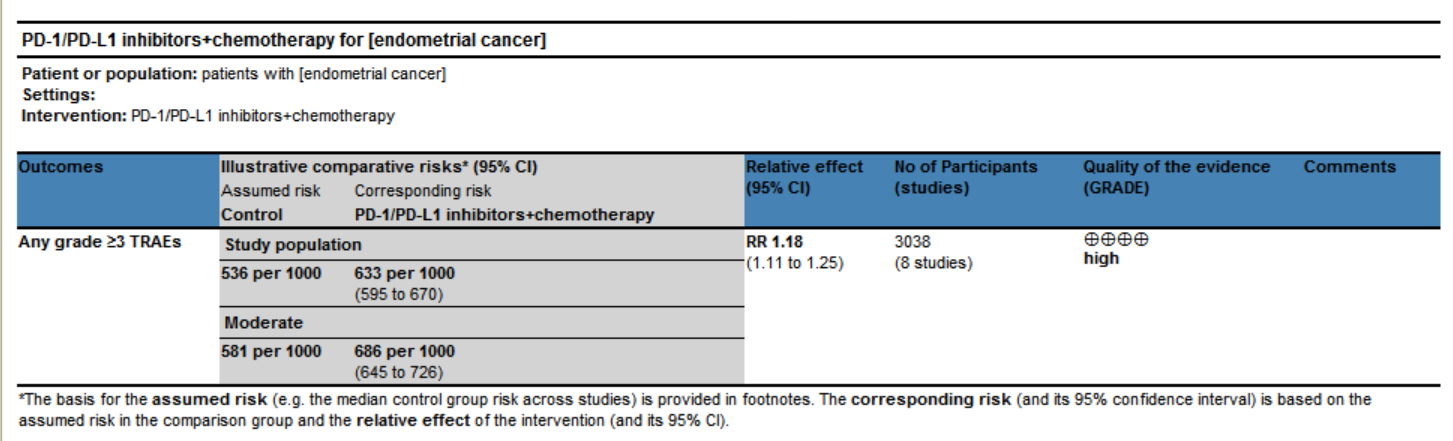


**eFigure 3.** Forest plot of Complete Response Rate


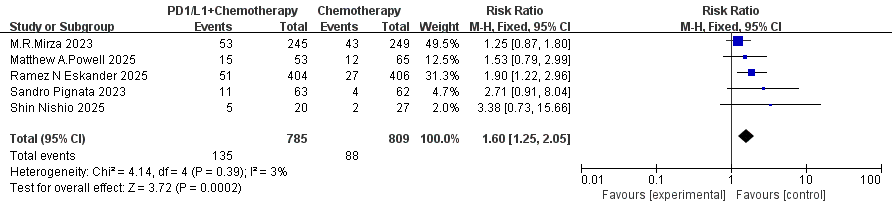


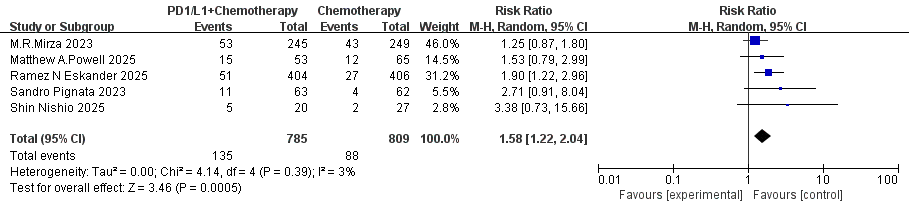


**eFigure 4.** Forest plot of Objective Response Rate


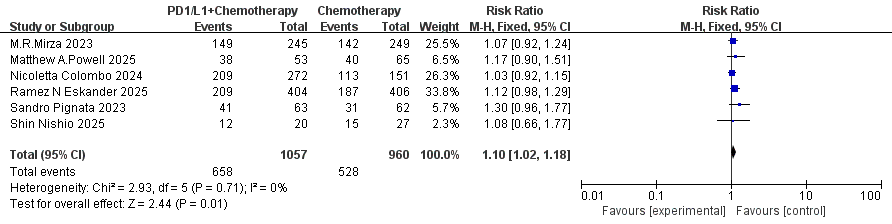


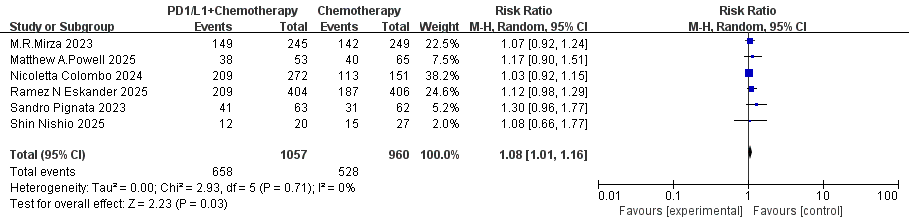


**eFigure 5.** Forest plot of Progressive Disease Rate


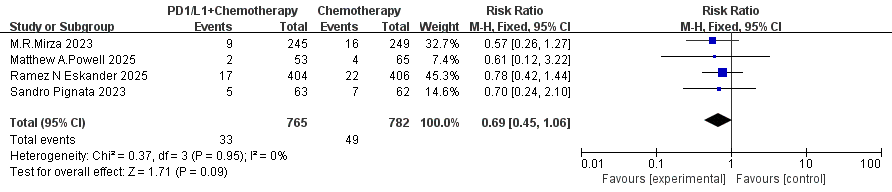


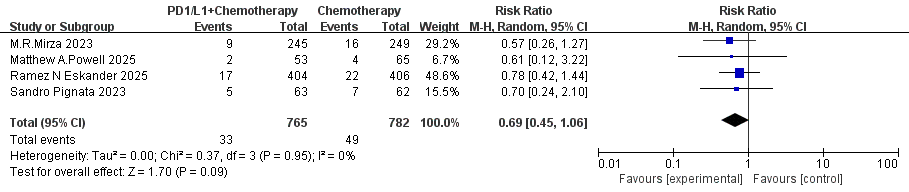


**eFigure 6.** Forest plot of Partial Response Rate


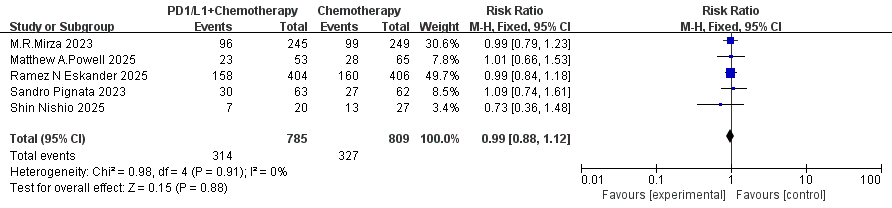


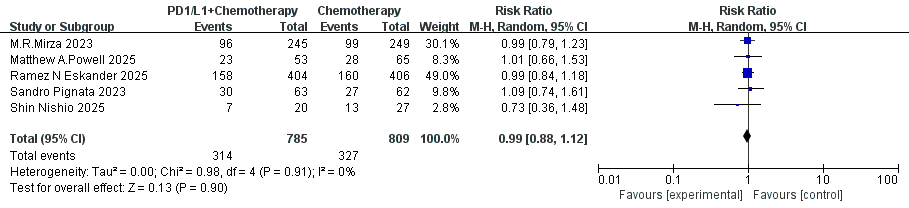


**eFigure 7.** Forest plot of Stable desease Rate


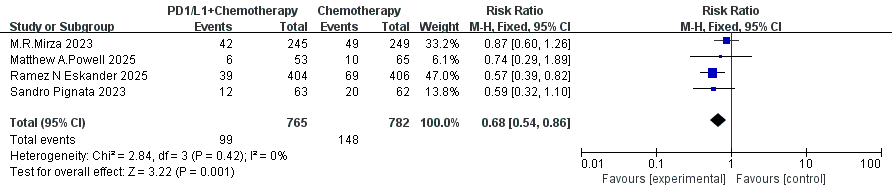


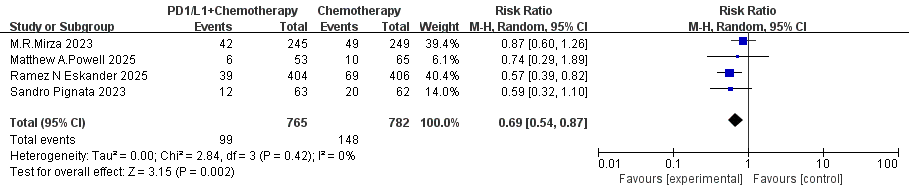


**eFigure 8.** Forest plot of Alopecia


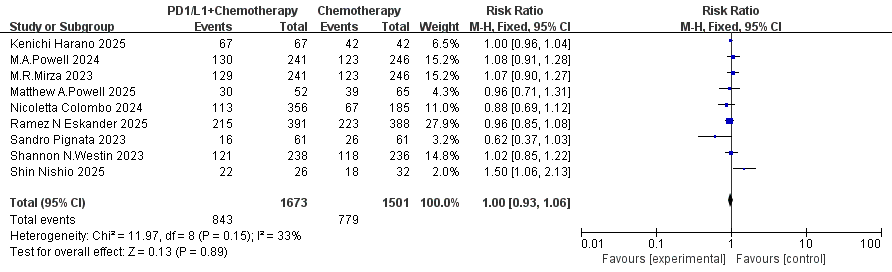


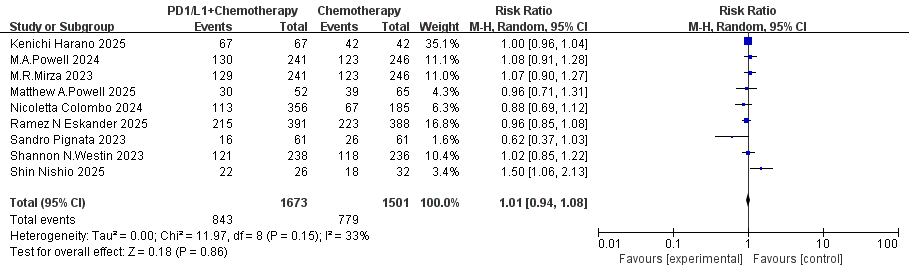


**eFigure 9.** Forest plot of Anemia


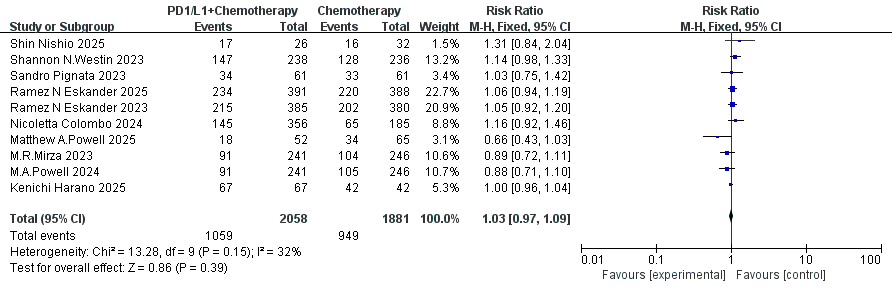


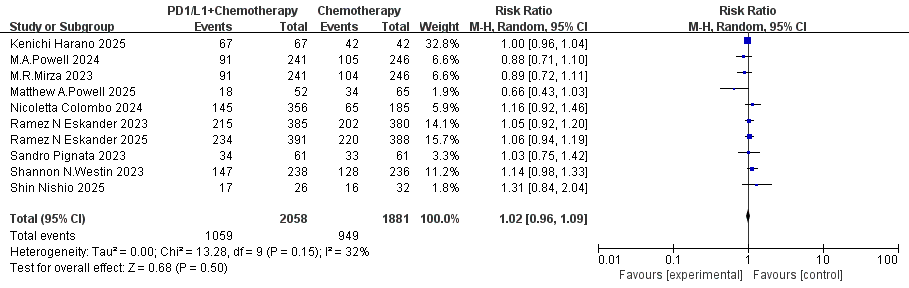


**eFigure 10.** Forest plot of Any grade ≥3 TRAE
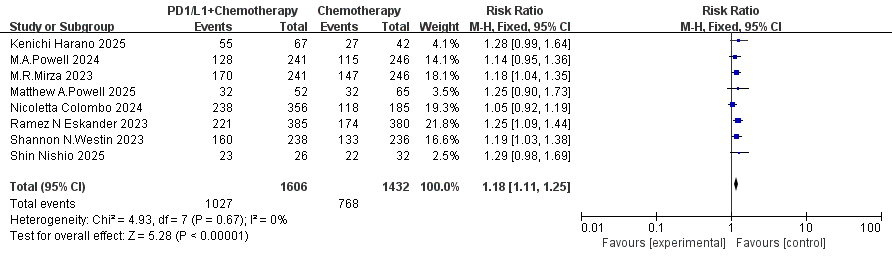


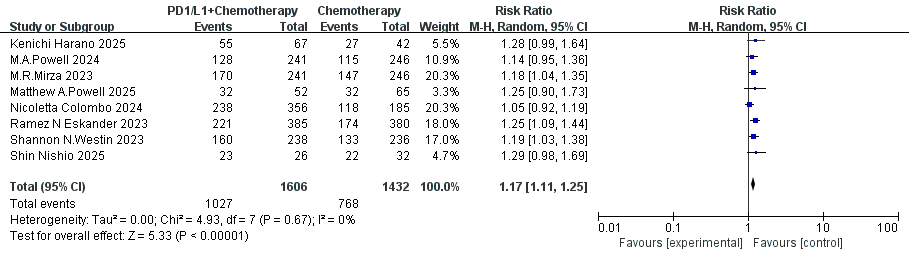


**eFigure 11.** Forest plot of Any TRAE


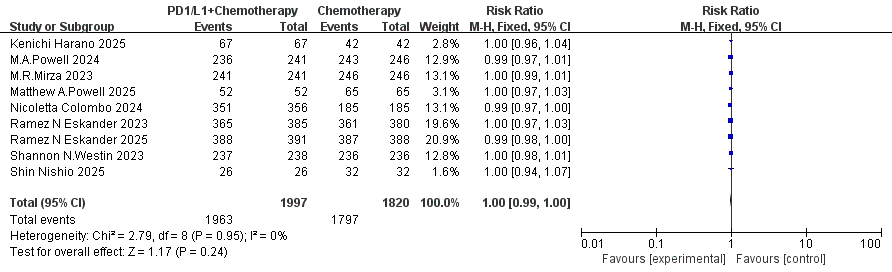


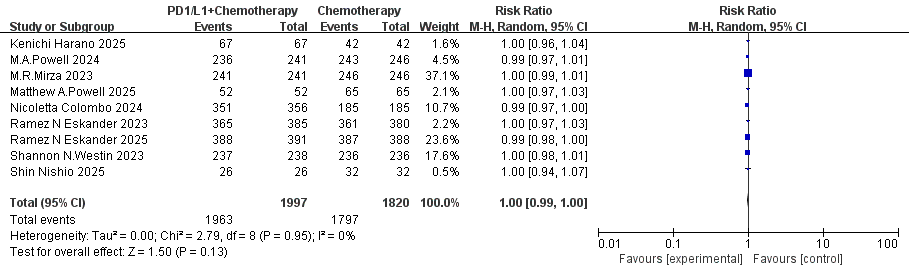


**eFigure 12.** Forest plot of Arthralgia
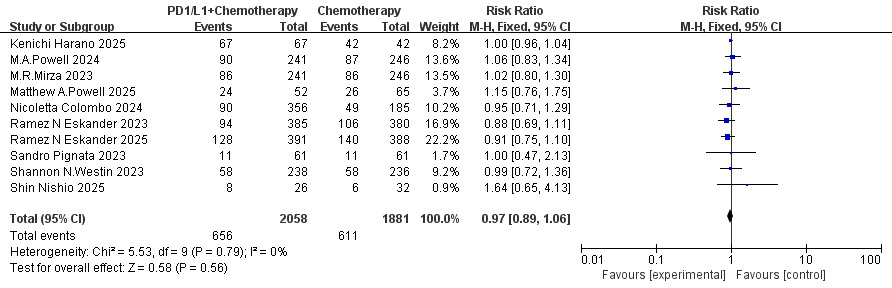


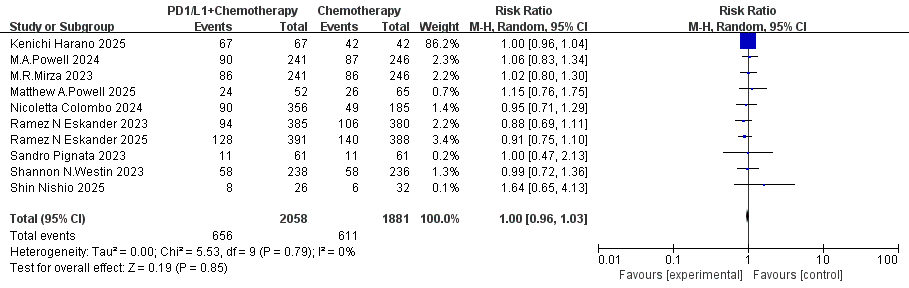


**eFigure 13.** Forest plot of Constipation


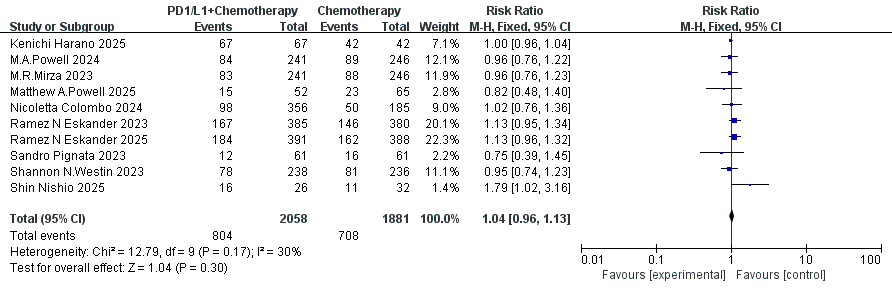


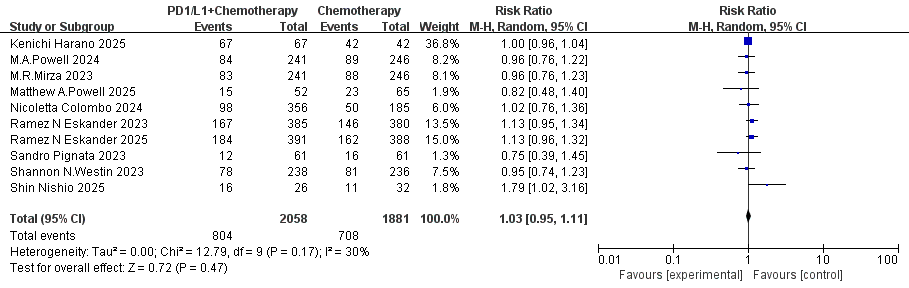


**eFigure 14.** Forest plot of Diarrhea


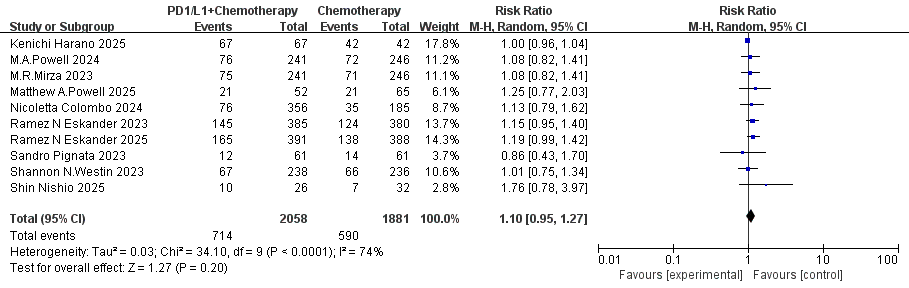


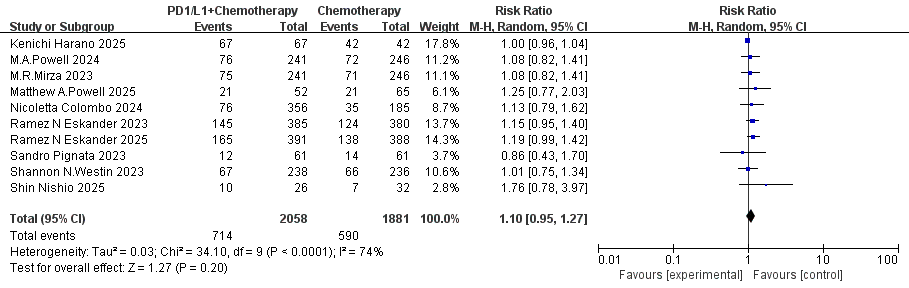


**eFigure 15.** Forest plot of Fatigue


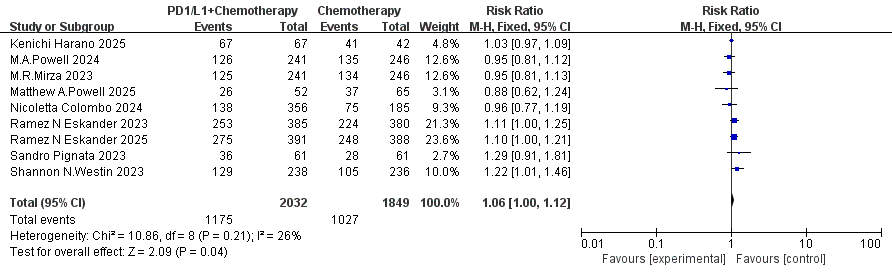


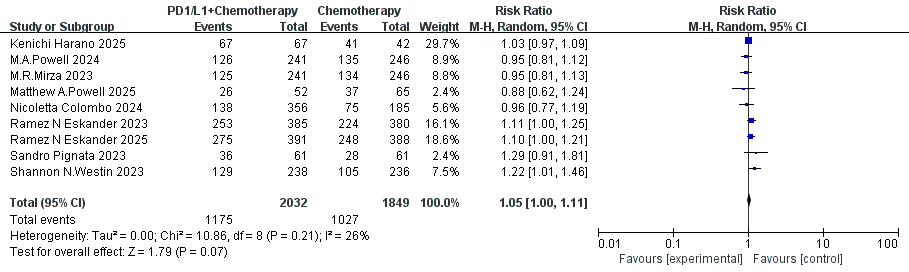


**eFigure 16.** Forest plot of Nausea


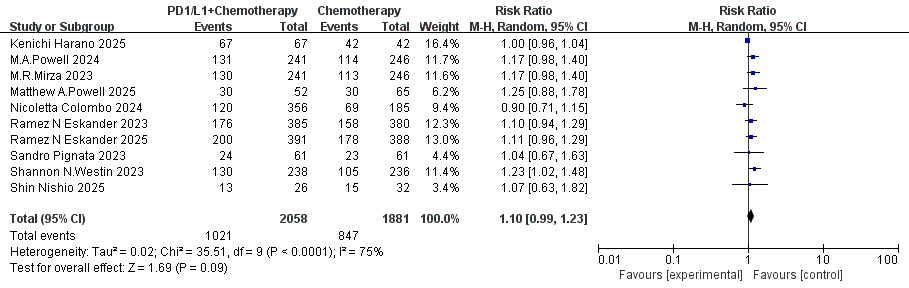


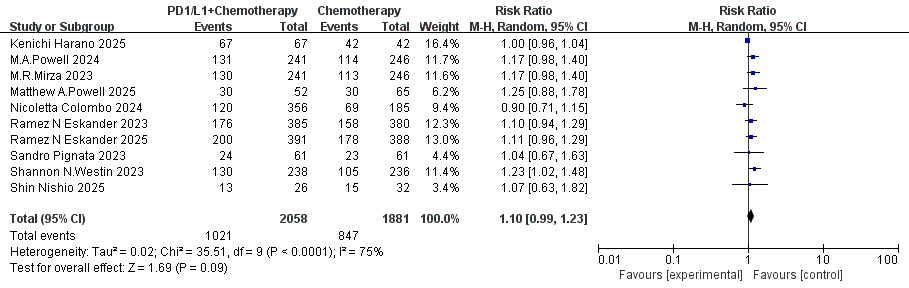


**eFigure 17.** Forest plot of Neuropathy peripheral


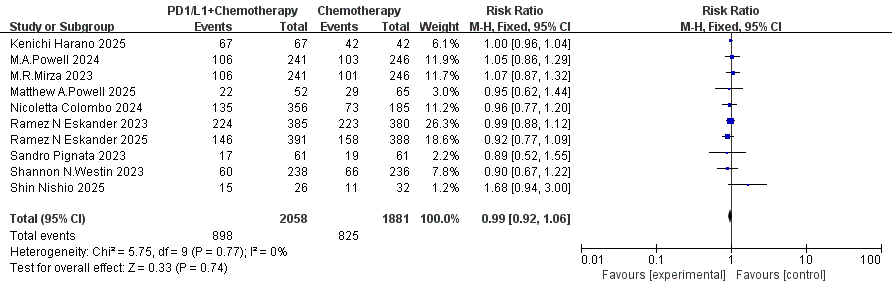


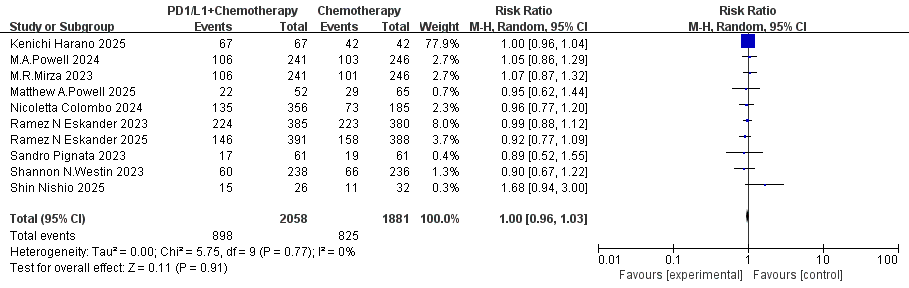


**eFigure 18.** Sensitivity analysis of Alopecia


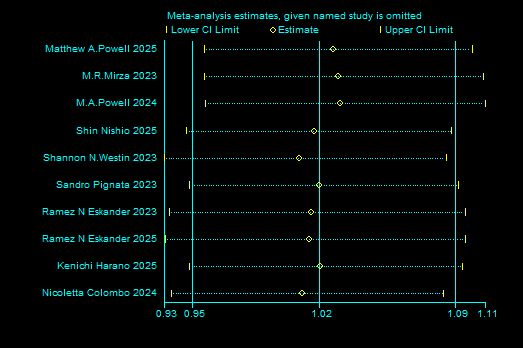


**eFigure 19.** Sensitivity analysis of Anemia


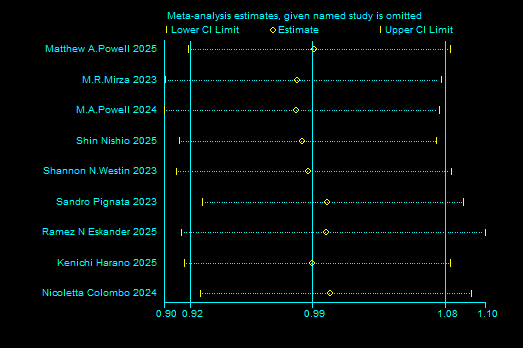


**eFigure 20.** Sensitivity analysis of Any grade ≥3 TRAE


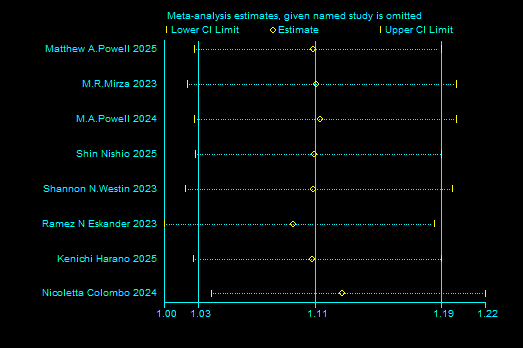


**eFigure 21.** Sensitivity analysis of Any TRAE


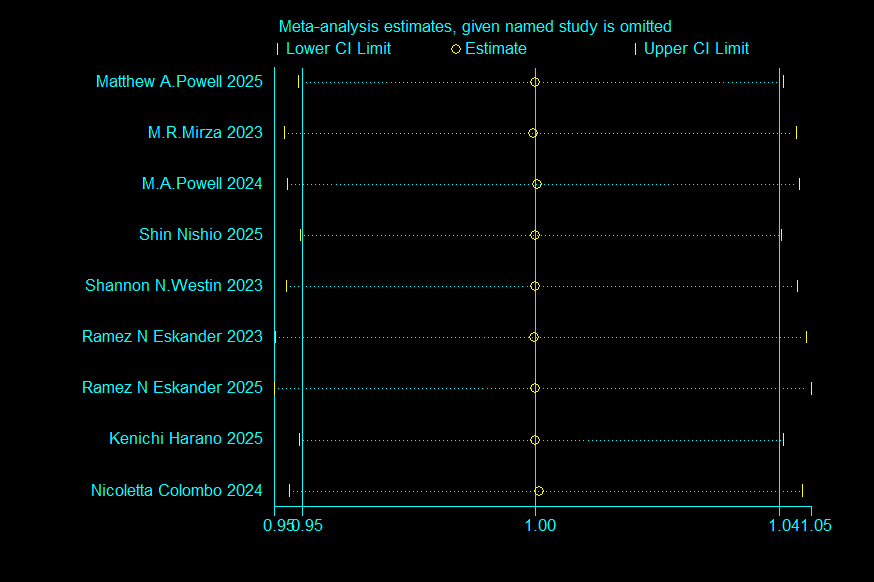


**eFigure 22.** Sensitivity analysis of Arthralgia


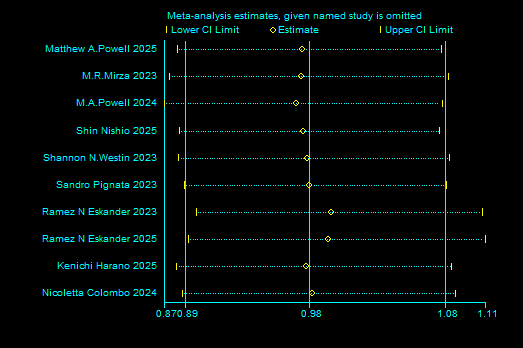


**eFigure 23.** Sensitivity analysis of Constipation
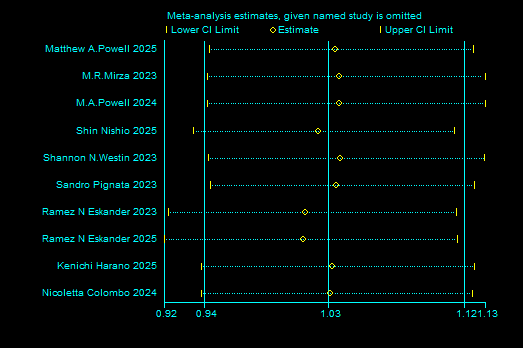


**eFigure 24.** Sensitivity analysis of Diarrhea


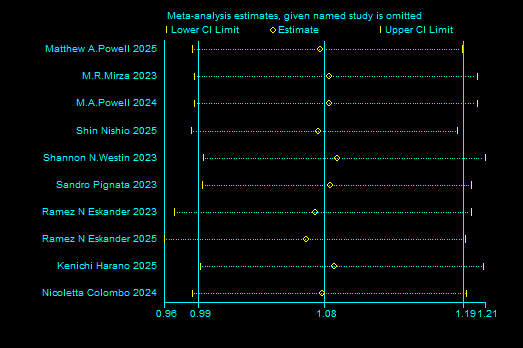


**eFigure 25.** Sensitivity analysis of Fatigue


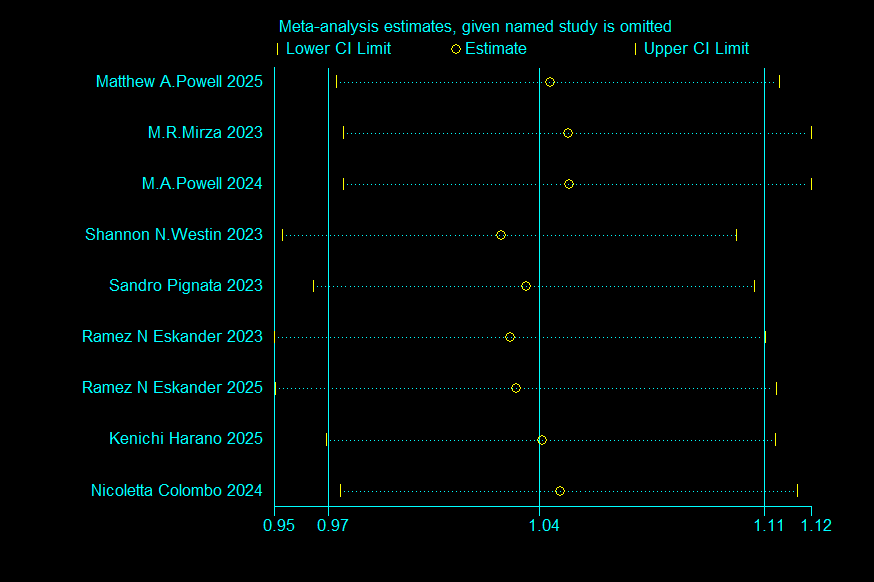


**eFigure 26.** Sensitivity analysis of Nausea


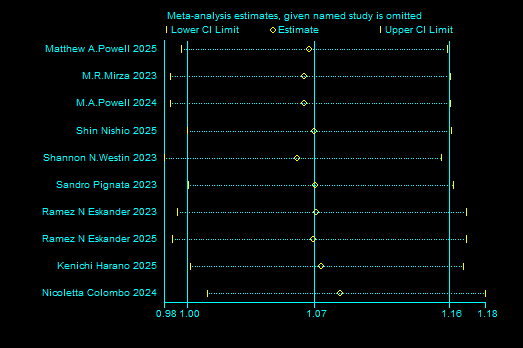


**eFigure 27.** Sensitivity analysis of Neuropathy peripheral


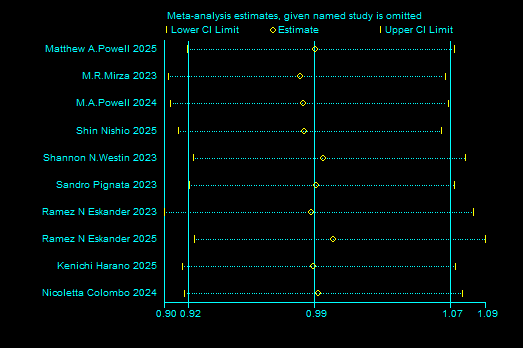


**eFigure 28.** Sensitivity analysis of ORR


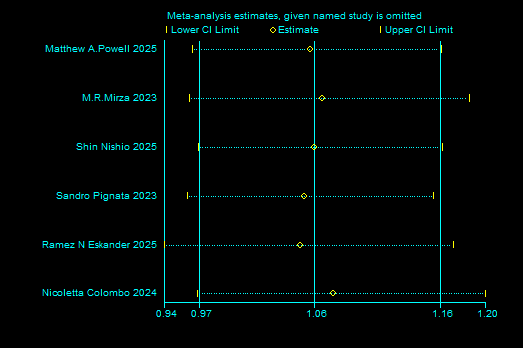


**eFigure 29.** Sensitivity analysis of CR


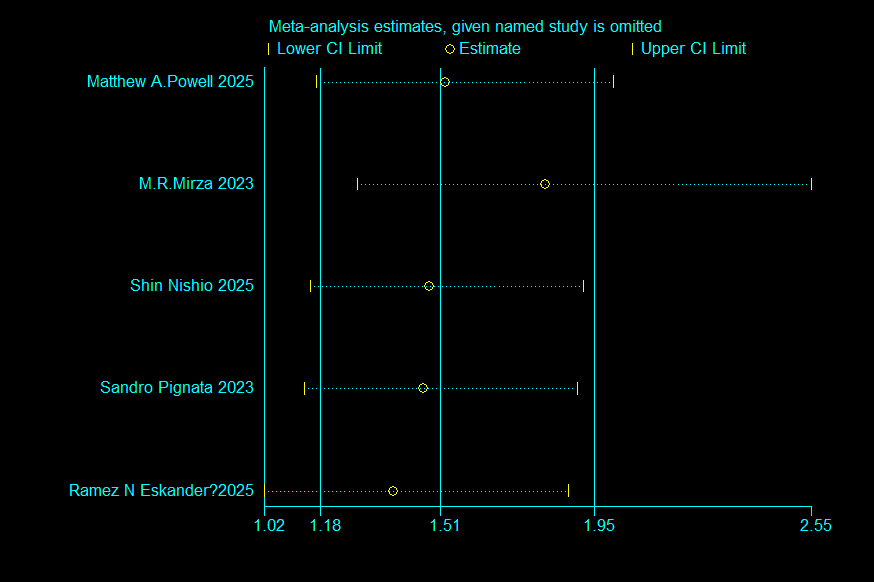


**eFigure 30.** Sensitivity analysis of PD


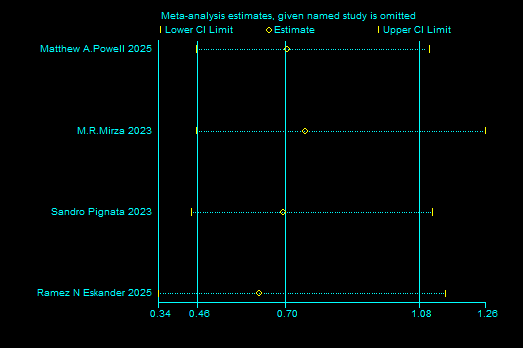


**eFigure 31.** Sensitivity analysis of PR


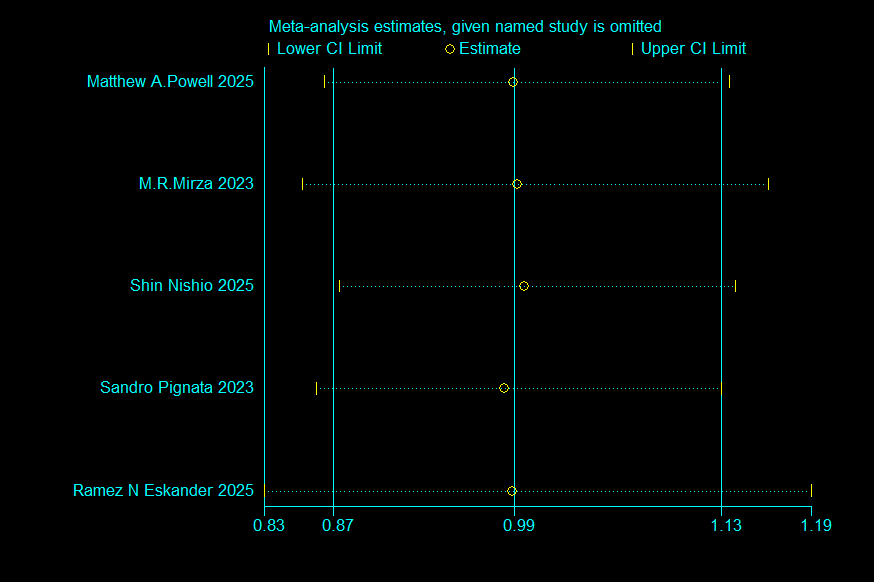


**eFigure 32.** Sensitivity analysis of SD


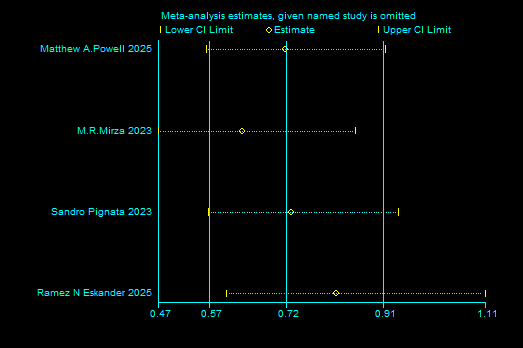


**eFigure 33.** Funnel plot of Complete Response Rate


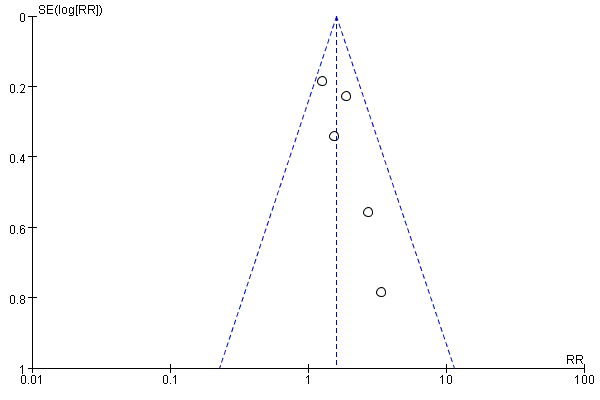


**eFigure 34.** Funnel plot of Objective Response Rate


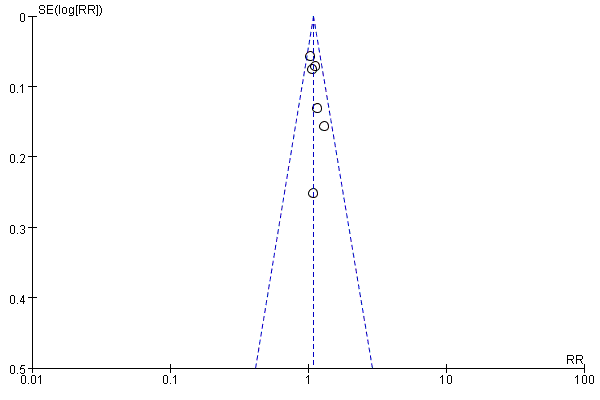


**eFigure 35.** Funnel plot of Progressive Disease Rate


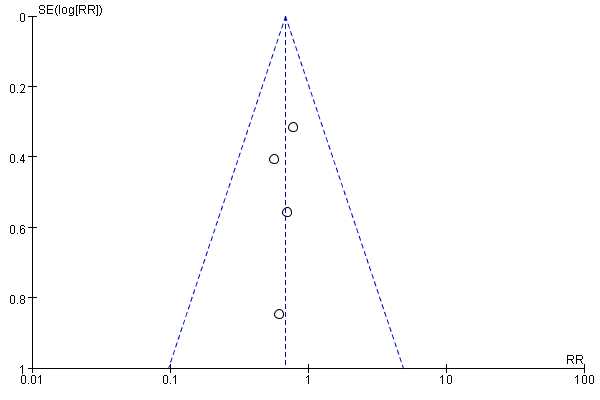


**eFigure 36.** Funnel plot of Partial Response Rate


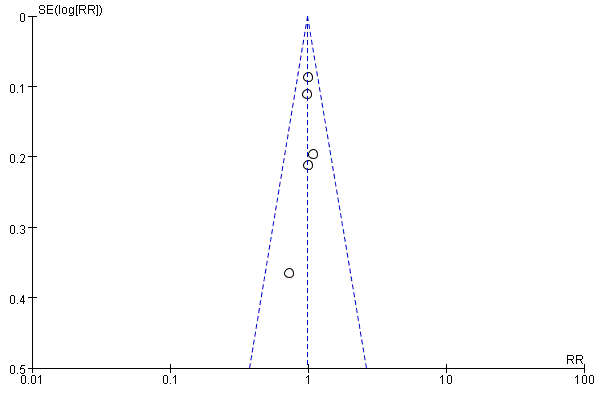


**eFigure 37.** Funnel plot of Stable desease Rate


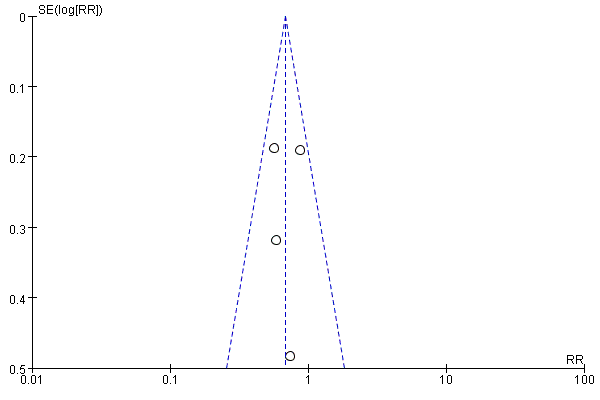


**eFigure 38.** Funnel plot of Alopecia


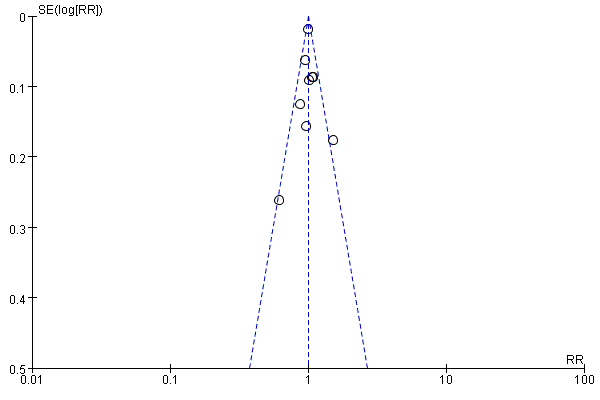


**eFigure 39.** Funnel plot of Anemia


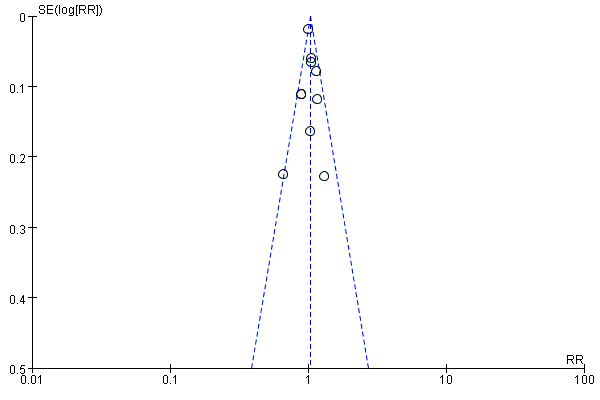


**eFigure 40.** Funnel plot of Any grade ≥3 TRAE


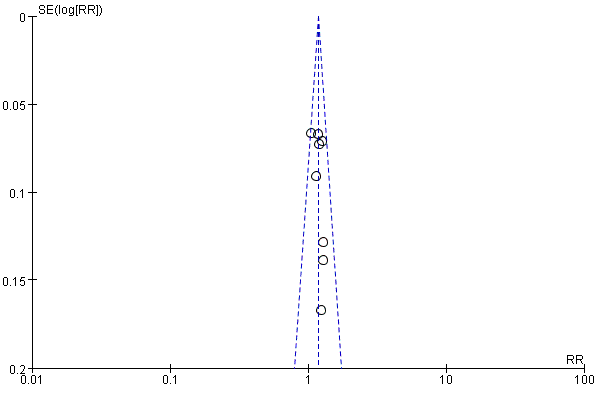


**eFigure 41.** Funnel plot of Any TRAE


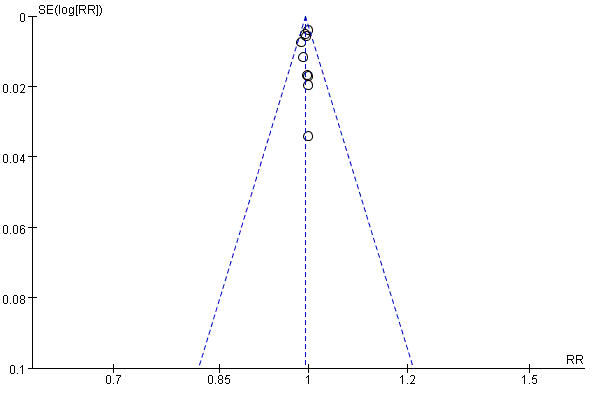


**eFigure 42.** Funnel plot of Arthralgia


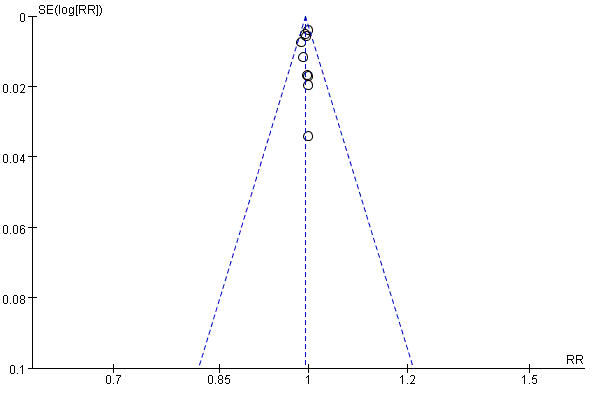


**eFigure 43.** Funnel plot of Constipation


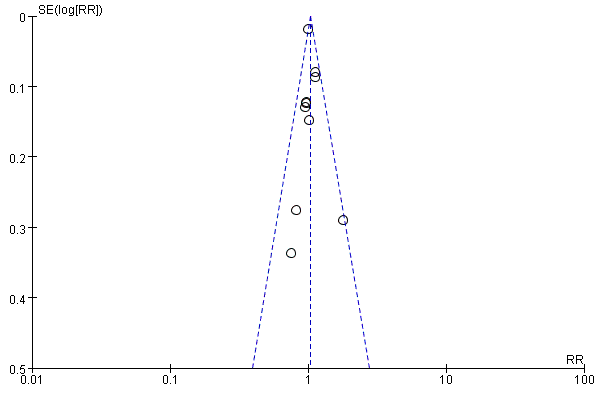


**eFigure 44.** Funnel plot of Diarrhea


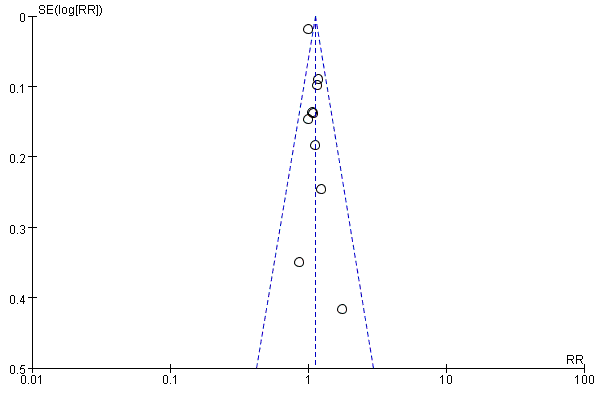


**eFigure 45.** Funnel plot of Fatigue


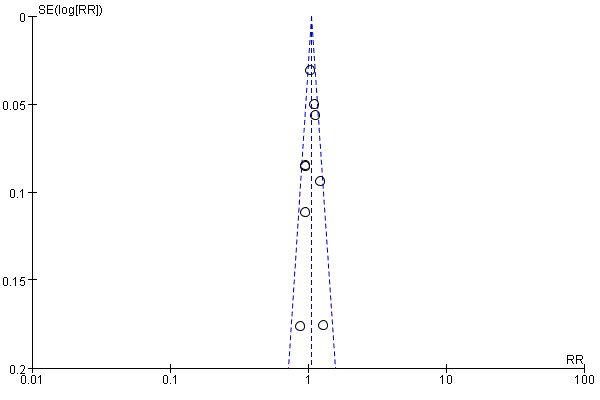


**eFigure 46.** Funnel plot of Nausea


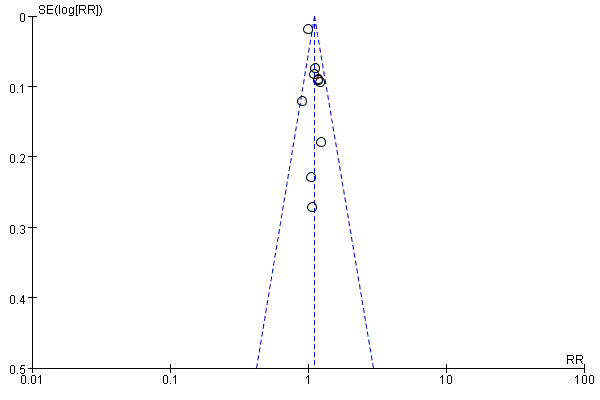


**eFigure 47.** Funnel plot of Neuropathy peripheral


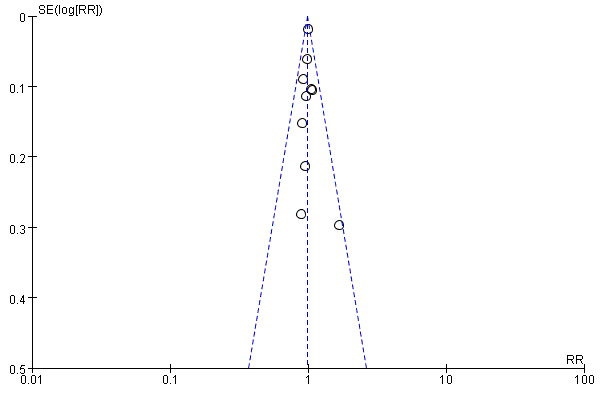


**eFigure 48.** Forest plot of PFS


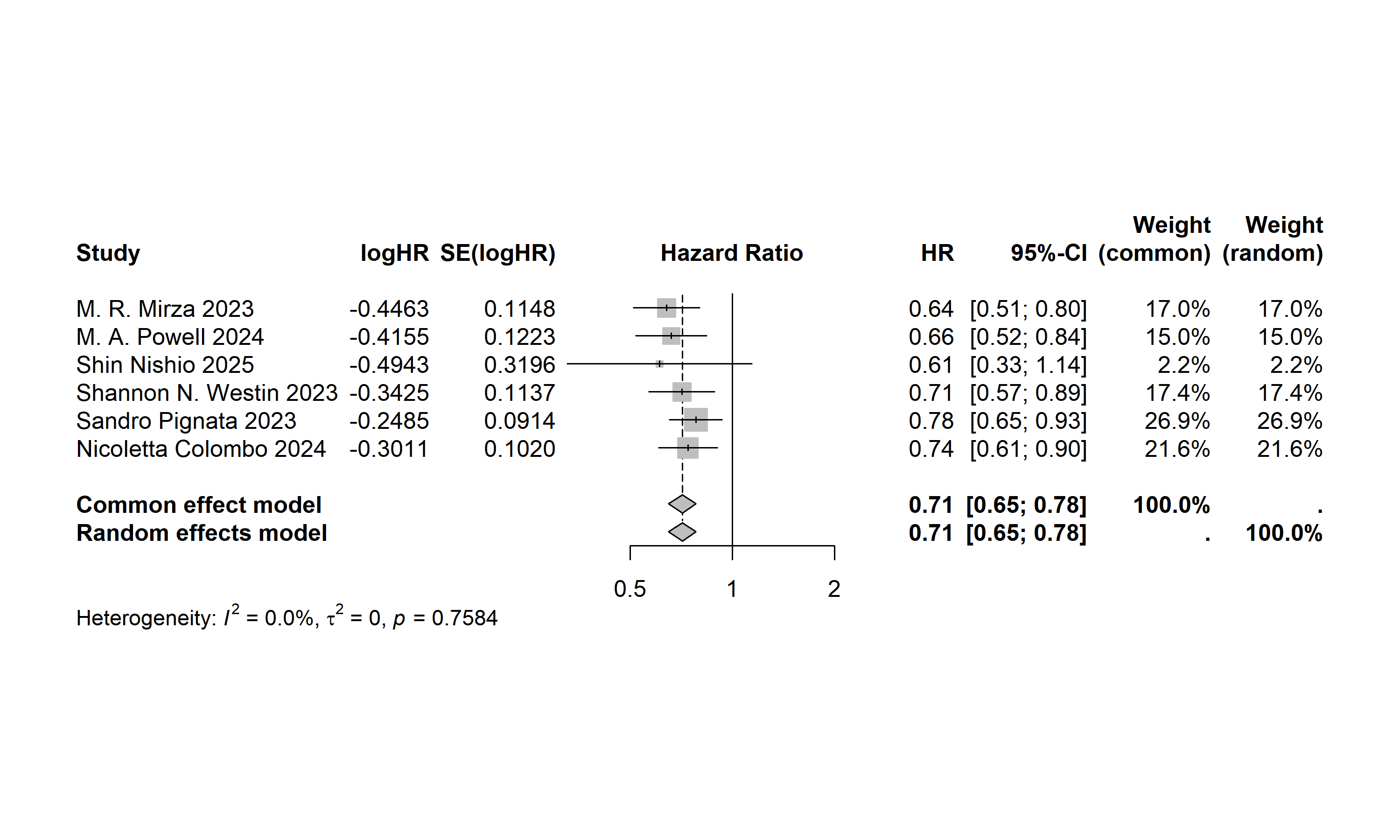


**eFigure 49.** Sensitivity analysis of PFS


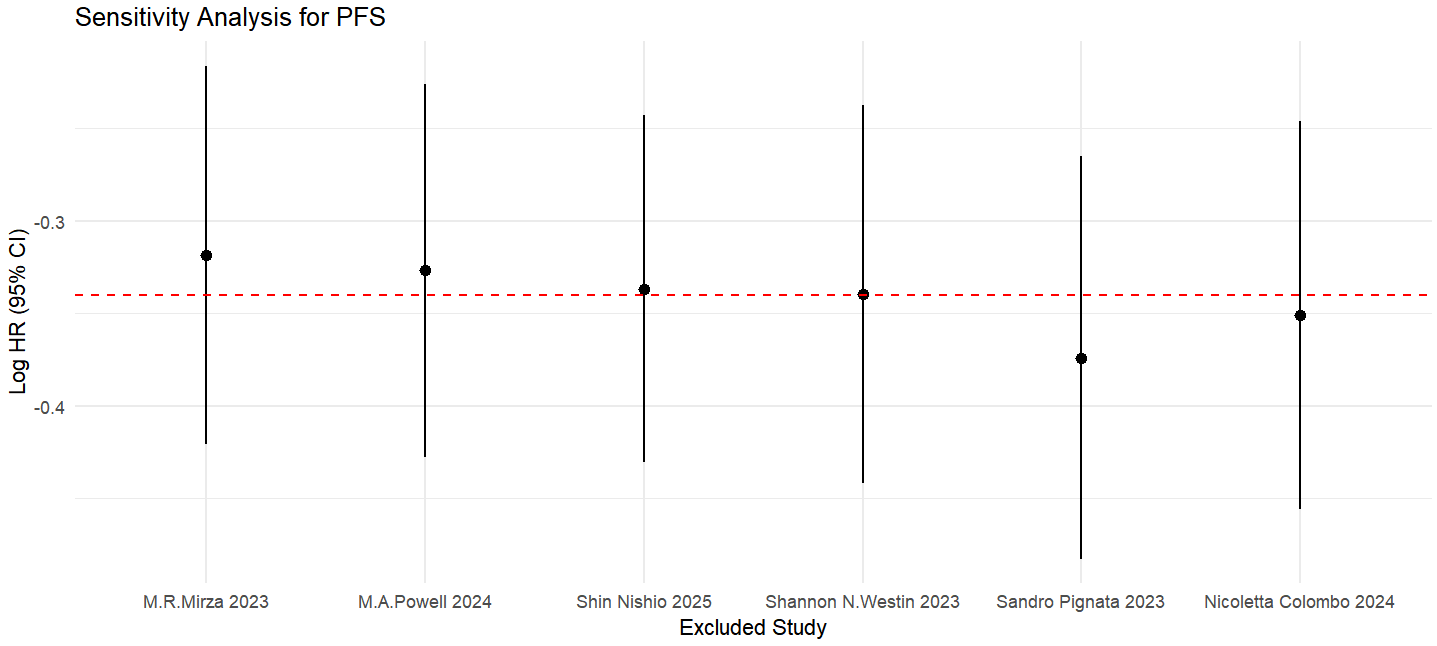


**eFigure 50.** Funnel plot of PFS


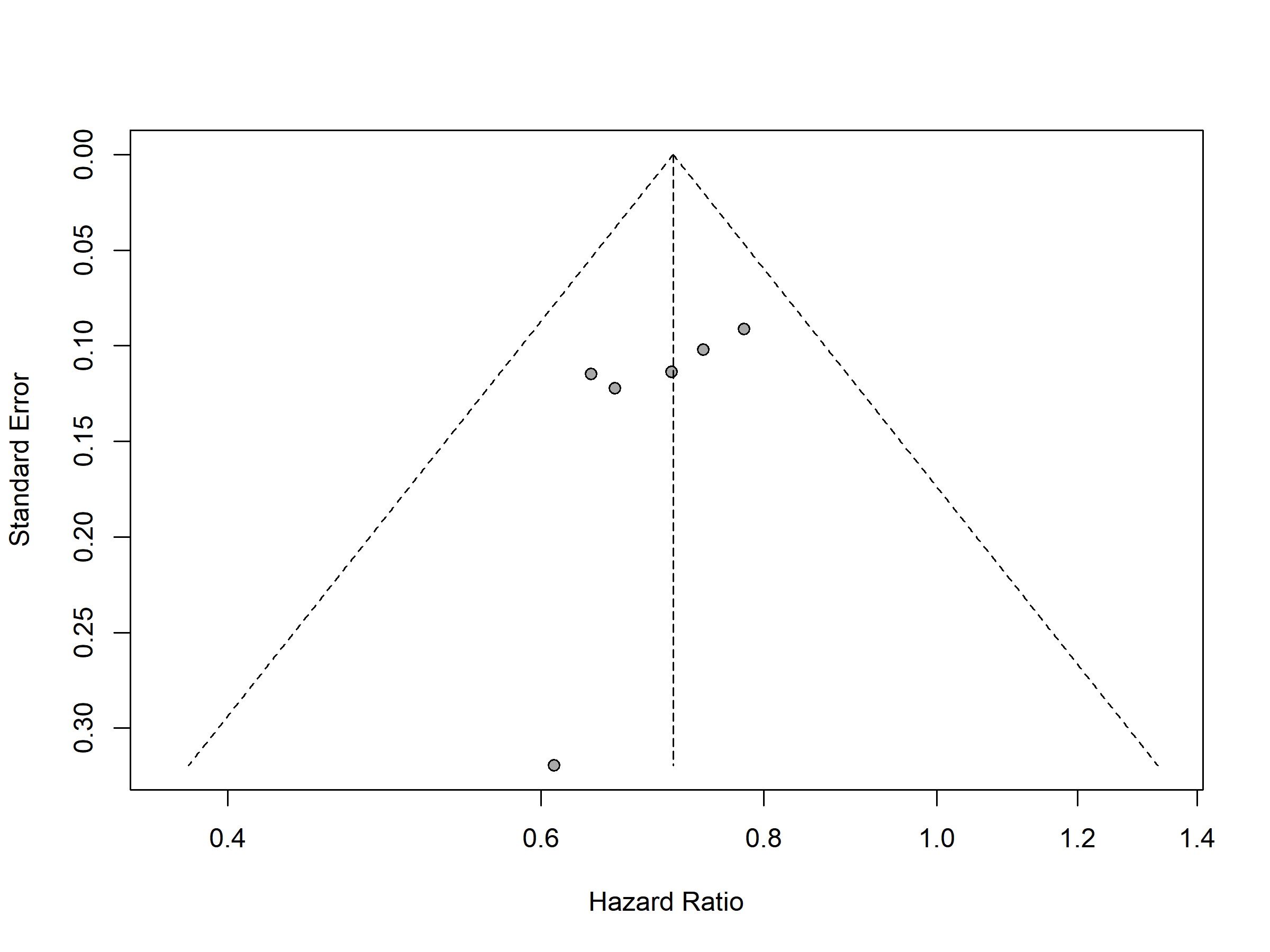


**eFigure 51.** Forest plot of OS


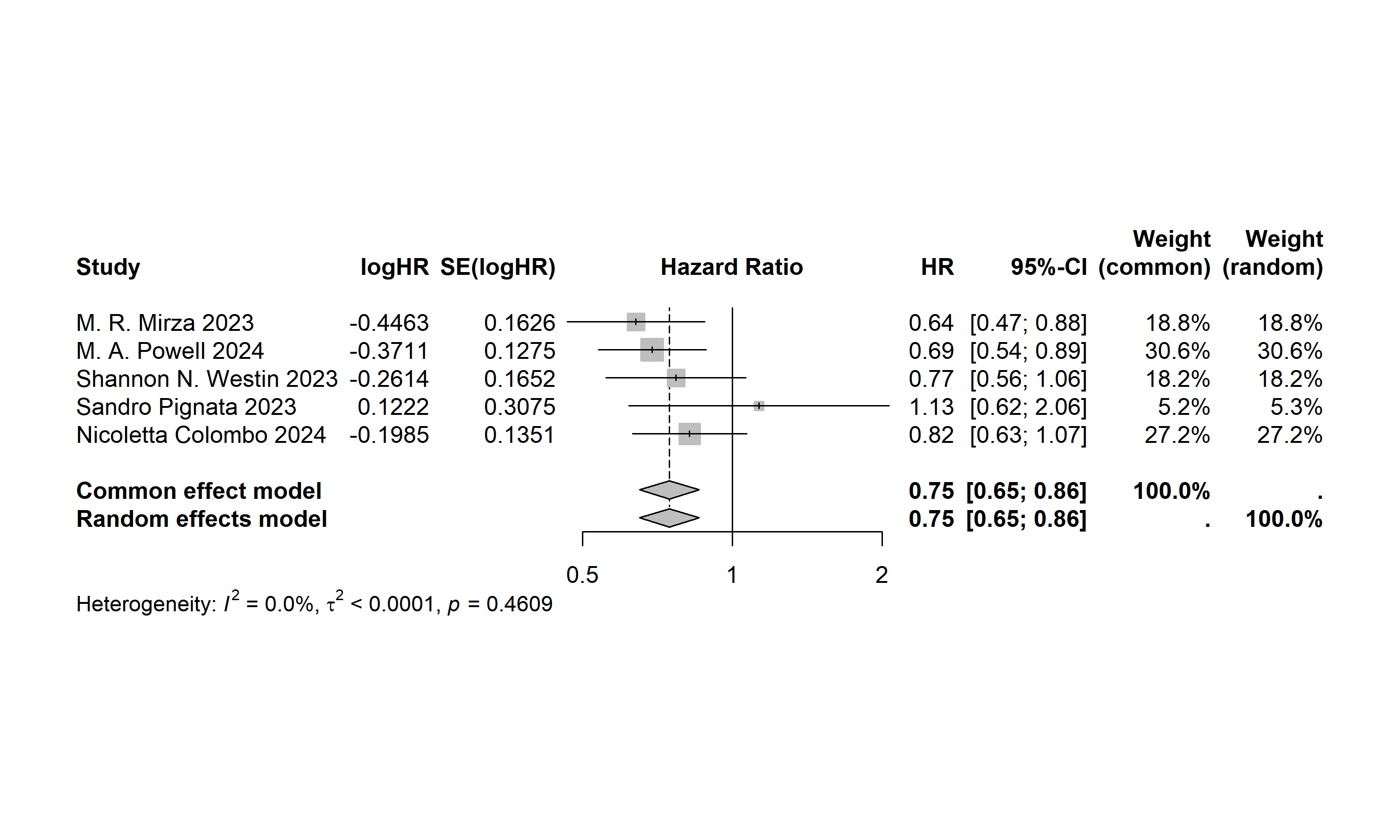


**eFigure 52.** Sensitivity analysis of OS


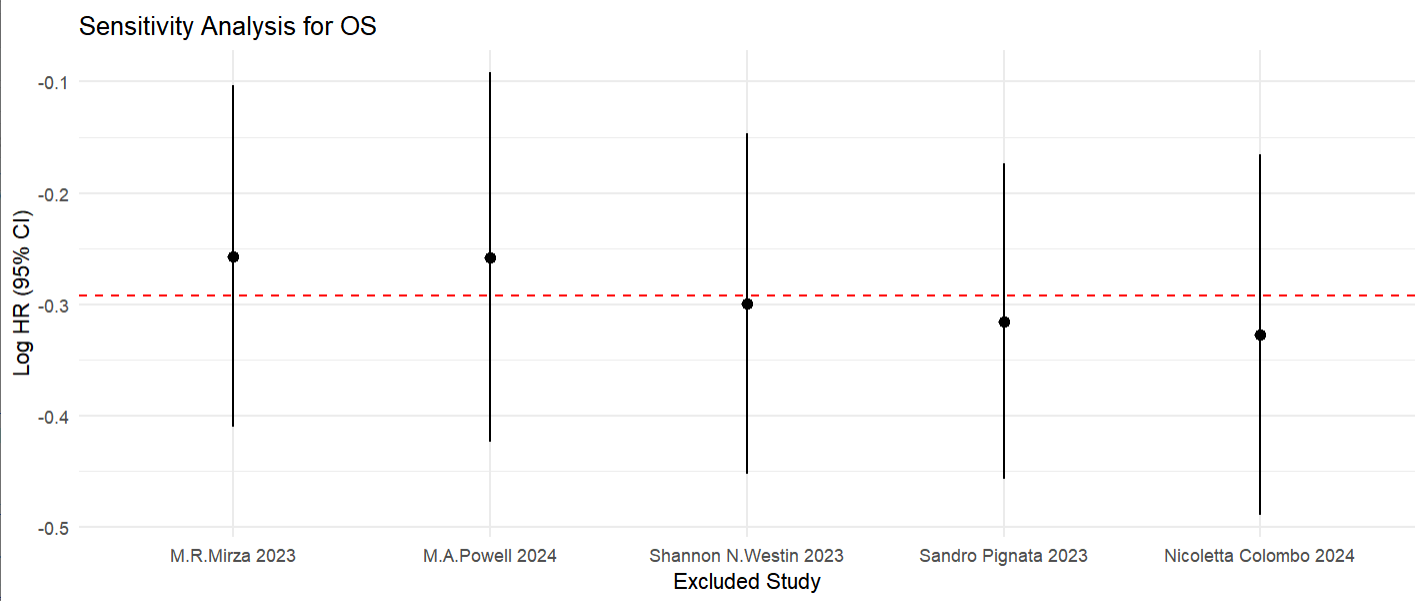


**eFigure 53.** Funnel plot of OS


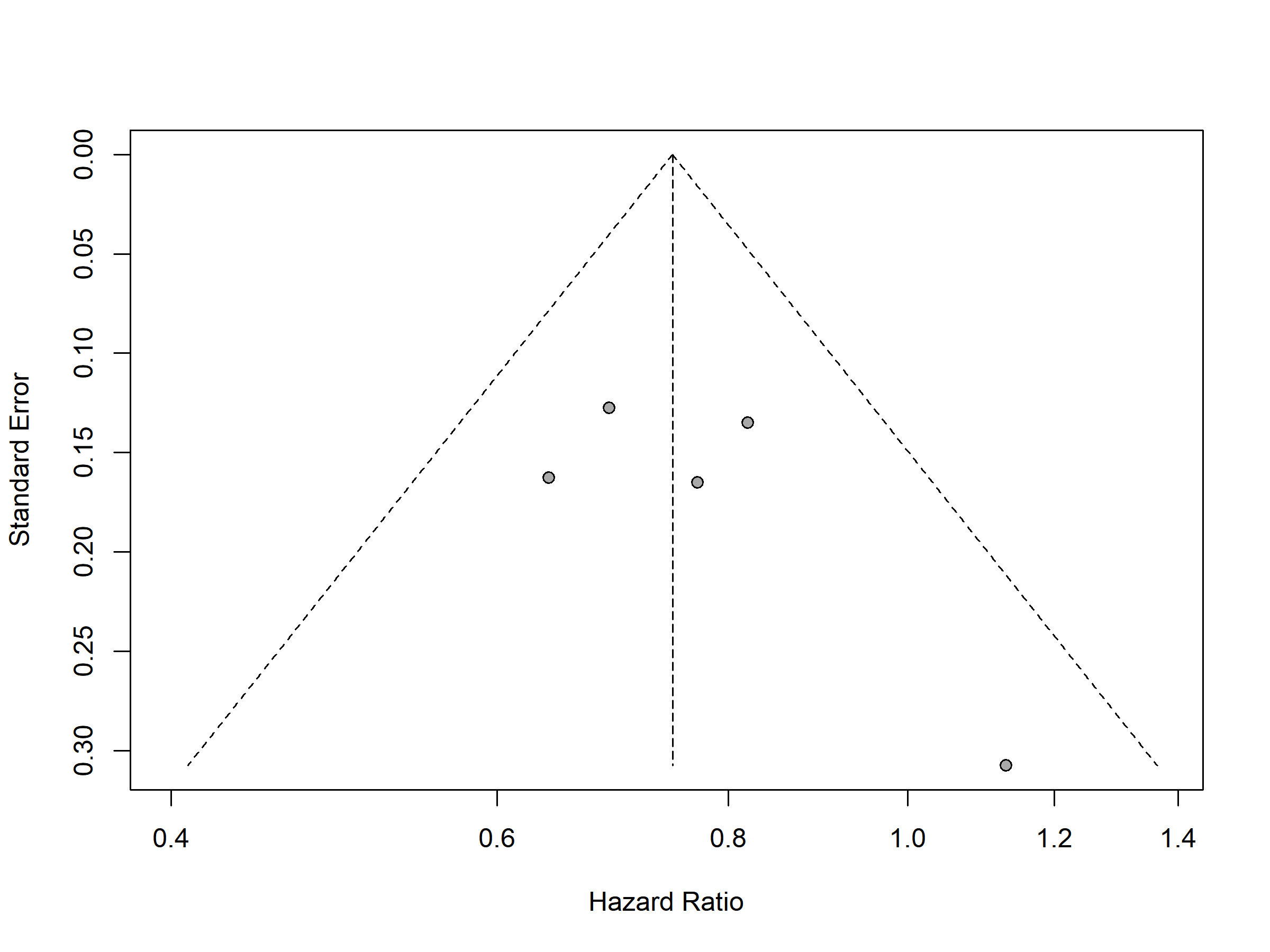


**Note:**Complete definitions of outcome

The definitions of all outcome events in this study were based on the original settings of the 10 included randomized controlled trials (RCTs) to ensure consistency with the primary studies. The complete definitions are as follows:

1. Overall Survival (OS)

Definition: The time from the date of randomization, when patients were assigned to the experimental group (PD-1/PD-L1 inhibitor combined with chemotherapy) or the control group (chemotherapy alone or other comparator regimens), to the date of death from any cause. Patients who were still alive at the data cutoff date were censored at the last known date they were confirmed to be alive.

Diagnostic criteria and basis for assessment: Determination was based on death certificates, electronic medical records, or clinical confirmation during follow-up. Deaths attributable to tumor progression, treatment-related adverse reactions, and other non-tumor-related causes were all included in the OS analysis.

2. Progression-Free Survival (PFS)

Definition: The time from the date of randomization to the date of first documented disease progression (PD) or death from any cause, whichever occurred first. Patients who were alive and had not experienced disease progression at the data cutoff date were censored at that date.

Diagnostic criteria and basis for assessment: Disease progression was determined according to the Response Evaluation Criteria in Solid Tumors (RECIST) version 1.1, specifically: a ≥20% increase in the sum of the longest diameters of target lesions compared to baseline, or the appearance of new lesions; unequivocal progression of non-target lesions or the appearance of new lesions; death was considered a progression event. All included studies adopted RECIST v1.1 for the assessment of progression in PFS.

3. Adverse Events (AEs)

Definition: Any abnormal physical symptoms, signs, or laboratory findings that occurred during study treatment (PD-1/PD-L1 inhibitor combined with chemotherapy or the comparator regimen) and within 30 days after the end of treatment, regardless of whether they were considered directly related to the study drug, were regarded as adverse events.

Diagnostic criteria and basis for assessment: Adverse events were graded according to the National Cancer Institute Common Terminology Criteria for Adverse Events (CTCAE) version adopted by each included study, with the specific grading criteria implemented per the respective version (Grade 1: mild adverse reaction, no intervention required; Grade 2: moderate adverse reaction, symptomatic treatment indicated; Grade 3: severe adverse reaction, treatment suspension and active intervention required; Grade 4: life-threatening reaction, urgent resuscitation required; Grade 5: death related to the adverse reaction).

In this study, the definitions of objective response rate (ORR), complete response (CR), partial response (PR), stable disease (SD), and progressive disease (PD) strictly followed the Response Evaluation Criteria in Solid Tumors (RECIST) version 1.1, consistent with the efficacy assessment criteria of the 10 included RCTs. The complete definitions are as follows:

1. Complete Response (CR)

Definition: Disappearance of all target lesions, disappearance of all non-target lesions, and normalization of tumor markers (if elevated at baseline) to the normal reference range, maintained for at least 4 weeks, with no appearance of new lesions.

Assessment details: (1) Target lesions: complete disappearance of all measurable target lesions (lesions with a longest diameter ≥10 mm determined at baseline), without any residual disease; (2) Non-target lesions: complete disappearance of all non-measurable non-target lesions (lesions with a longest diameter <10 mm); (3) Tumor markers: if tumor markers (e.g., CA125) were above the normal reference range at baseline, they must have returned to normal; (4) Duration: the response status had to be maintained for ≥4 weeks to exclude false-positive response due to short-term fluctuation.

2. Partial Response (PR)

Definition: A ≥30% decrease in the sum of the longest diameters of target lesions compared to baseline, with no appearance of new lesions and no unequivocal progression of non-target lesions, maintained for at least 4 weeks.

Assessment details: (1) Target lesions: the sum of the longest diameters of all target lesions after treatment decreased by ≥30% relative to baseline, calculated using the same imaging modality (e.g., CT, MRI) to ensure measurement consistency; (2) Non-target lesions: no unequivocal progression (no new lesions and no marked enlargement of pre-existing non-target lesions); (3) Duration: response must have persisted for ≥4 weeks to avoid misclassification due to transient treatment effects; (4) Note: if the sum of target lesion diameters decreased but did not reach 30%, or decreased ≥30% but lasted less than 4 weeks, PR was not assigned.

3. Stable Disease (SD)

Definition: Neither sufficient shrinkage to qualify for PR (decrease <30%) nor sufficient increase to qualify for PD (increase <20%) in the sum of the longest diameters of target lesions compared to baseline, with no appearance of new lesions and no unequivocal progression of non-target lesions.

Assessment details: (1) Target lesions: the change in the sum of the longest diameters relative to baseline ranged between -30% and +20%, without marked enlargement or shrinkage; (2) Non-target lesions: no unequivocal progression, no new lesions; (3) Special circumstances: if the baseline sum of target lesion diameters was small and a slight post-treatment increase (<20%) was observed, but no new lesions and no progression of non-target lesions occurred, the status was still classified as SD; (4) Duration: no fixed duration requirement was stipulated, but the assessment had to be combined with the follow-up interval to rule out measurement errors.

4. Progressive Disease (PD)

Definition: PD was defined by the occurrence of any of the following: (1) a ≥20% increase in the sum of the longest diameters of target lesions compared to baseline; (2) the appearance of any new tumor lesion(s), irrespective of changes in target lesions; (3) unequivocal progression of non-target lesions (marked enlargement of pre-existing non-target lesions or the appearance of new non-target lesions).

Assessment details: (1) Target lesions: the sum of the longest diameters increased by ≥20% relative to baseline, and the absolute increase was required to be ≥5 mm (if the baseline sum was small and the increase was ≥20% but the absolute value was <5 mm, PD was not immediately designated; confirmation by subsequent follow-up was necessary); (2) New lesions: any newly appearing tumor lesion (including metastasis outside the primary site) had to be confirmed by imaging (CT, MRI, etc.) to exclude artifacts or benign lesions; (3) Non-target lesions: marked enlargement of pre-existing non-target lesions or the appearance of new non-target lesions was considered progression of non-target disease.

5. Objective Response Rate (ORR)

Definition: The proportion of patients who achieved a best overall response of complete response (CR) or partial response (PR) among the total enrolled patients, serving as one of the core endpoints for evaluating antitumor efficacy.

Calculation and basis of assessment: ORR = (Number of patients with CR + Number of patients with PR) / Total number of enrolled patients × 100%. The determination of CR and PR strictly adhered to the above definitions and RECIST v1.1 criteria. The calculation method and assessment criteria of ORR were consistent across all included studies without any discrepancy.
